# Ethnomedicinal Usage, Phytochemistry and Pharmacological Potential of *Solanum surattense* Burm. f.

**DOI:** 10.3390/ph17070948

**Published:** 2024-07-15

**Authors:** Kamrul Hasan, Shabnam Sabiha, Nurul Islam, João F. Pinto, Olga Silva

**Affiliations:** 1 Research Institute for Medicines (iMed.ULisboa), Faculty of Pharmacy, Universidade de Lisboa, 1649-003 Lisbon, Portugal; mdkamrul@edu.ulisboa.pt (K.H.); s.sabiha@edu.ulisboa.pt (S.S.); jfpinto@ff.ul.pt (J.F.P.); 2 Department of Zoology, Faculty of Biological Sciences, University of Rajshahi, Rajshahi 6205, Bangladesh; n_islamm@yahoo.com

**Keywords:** anti-cancer, anti-inflammatory, ethnomedicine, ethnopharmacology, natural products, *Solanum surattense*, steroidal saponin, steroidal alkaloid

## Abstract

*Solanum surattense* Burm. f. is a significant member of the *Solanaceae* family, and the *Solanum* genus is renowned for its traditional medicinal uses and bioactive potential. This systematic review adheres to PRISMA methodology, analyzing scientific publications between 1753 and 2023 from B-on, Google Scholar, PubMed, Science Direct, and Web of Science, aiming to provide comprehensive and updated information on the distribution, ethnomedicinal uses, chemical constituents, and pharmacological activities of *S. surattense*, highlighting its potential as a source of herbal drugs. Ethnomedicinally, this species is important to treat skin diseases, piles complications, and toothache. The fruit was found to be the most used part of this plant (25%), together with the whole plant (22%) used to treat different ailments, and its decoction was found to be the most preferable mode of herbal drug preparation. A total of 338 metabolites of various chemical classes were isolated from *S. surattense*, including 137 (40.53%) terpenoids, 56 (16.56%) phenol derivatives, and 52 (15.38%) lipids. Mixtures of different parts of this plant in water–ethanol have shown in vitro and/or in vivo antioxidant, anti-inflammatory, antimicrobial, anti-tumoral, hepatoprotective, and larvicidal activities. Among the metabolites, 51 were identified and biologically tested, presenting antioxidant, anti-inflammatory, and antitumoral as the most reported activities. Clinical trials in humans made with the whole plant extract showed its efficacy as an anti-asthmatic agent. Mostly steroidal alkaloids and triterpenoids, such as solamargine, solanidine, solasodine, solasonine, tomatidine, xanthosaponin A–B, dioscin, lupeol, and stigmasterol are biologically the most active metabolites with high potency that reflects the new and high potential of this species as a novel source of herbal medicines. More experimental studies and a deeper understanding of this plant must be conducted to ensure its use as a source of raw materials for pharmaceutical use.

## 1. Introduction

*Solanum surattense* Burm. f. is an important species of the Nightshade family *Solanaceae* and genus *Solanum* L., which is the most representative and largest genus comprising 1235 accepted species [1]. 

There was a debate in the past concerning the *Solanum surattense* species name, after which this species was named *Solanum virginianum* L. by Linnaeus (1753) [2]. After that, Burmanii (1768) described it and named it *S. surattense* [3], and *S. surattense* by Scharder and Wendland (1795) [4] based on *S. virginianum*. It has 16 synonyms, but among them, *S. surattense* was mostly used as a synonym [5,6,7]. However, *S. surattense* is now stated as the legitimate taxonomic name [8], and *S. virginianum* is used as the basionym of this species. This species is known by different local names in different countries (Table 1).

**Figure 1:**
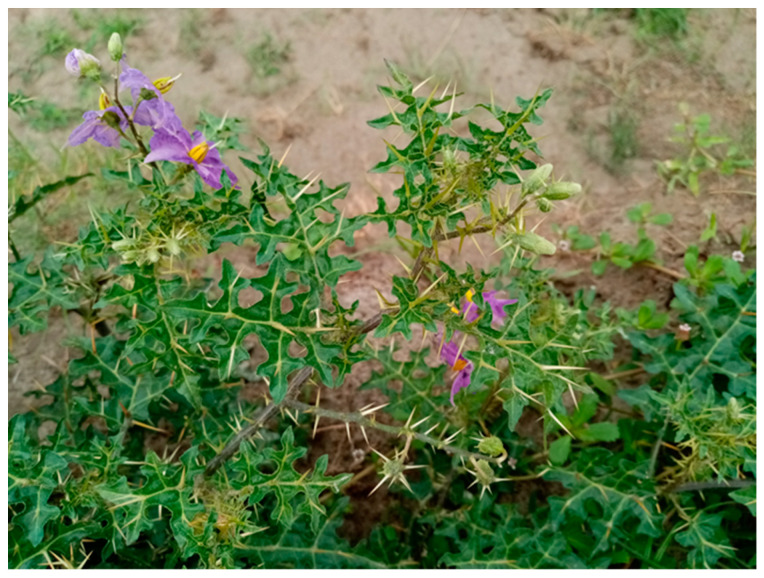
General picture of *S. surattense*.

*S. surattense* is widely distributed in tropical and subtropical parts of Southeast Asia [12]. It is a very common species in Bangladesh and spread all over the country [9]. Morphologically, this species is a prickly, diffuse, perennial herb with procumbent branches, bearing numerous, compressed, straight, and bright yellow prickles (Figure 1). The leaf is very prickly, deeply pinnately lobed with a sinuous outline to the lobes, and very unequal at the base. The flowers are pentamerous, prickly, and purple to bluish-purple. The fruit is a spherical berry, white with green marking when young but light yellow or whitish when ripe [9,13]. The preferable habitat of this species is dry, sunny places, wastelands, along roadsides, and degraded forest areas.

*S. surattense* is a significant *Solanum* species reported for its medicinal properties. In accordance, *S. surattense* is described as being used in traditional medicine in various Oriental regions for treating different ailments. In India and Pakistan, it is commonly used for addressing respiratory issues such as asthma and cough, skin diseases, gastrointestinal disorders, and urinary problems. Its fruit, root, and whole plant are primarily used for the treatment of these health problems, in the form of decoctions, powders, oral and topical administration, and topical semi-solid formulations such as pastes.

Antioxidant, anti-inflammatory, antimicrobial, anti-tumoral, hepatoprotective, and larvicidal biological activities, were demonstrated to different *S. surattense* plant parts traditional herbal preparations through both in vitro and/or in vivo assays and the marker secondary metabolites responsible or involved in some of these activities identified. Two clinical trials made in humans have confirmed the usefulness of this plant species as an anti-asthmatic agent, reinforcing its potential for therapeutic applications.

Although some of the literature reviews have already been published [14,15,16,17,18] on *S. surattense*, there are compelling reasons supporting the work hereby presented:

- Reviewing the current scientific research on the species and evaluate the extent of the knowledge that has been published in a broad range of reputable scientific databases;
- Representing the ethnomedicinal potential of *Solanum surattense* and validating the knowledge scientifically;
- Highlight the identification, characterization, and potentialities of isolated secondary metabolites in terms of drug discovery and development;
- Documentation of more up-to-date information concerning the pharmacological effects of the species. Conclusively, by analyzing the gaps in prior research, providing a detailed account of the species’ ethnomedicinal uses, chemical constituents, and pharmacological properties. This will enable researchers and medical professionals to have access to the most recent scientific evidence, facilitating the development of new herbal medications and promoting the safe and effective use of these traditional medicinal plants.

Scientific data for this review were meticulously collected from several reputable databases, including B-on, Google Scholar, PubMed, Science Direct, and Web of Science. The search spanned all scientific publications published between 1753 and 2023. The specific thesaurus terms used in the search were “*Solanum surattense*”, “*Solanum xanthocarpum*”, “ethnomedicinal value”, “traditional use”, “phytochemical analysis”, and “pharmacological activity”. 

## 2. Results and Discussion

### 2.1. Selection of the Information

**Figure 2:**
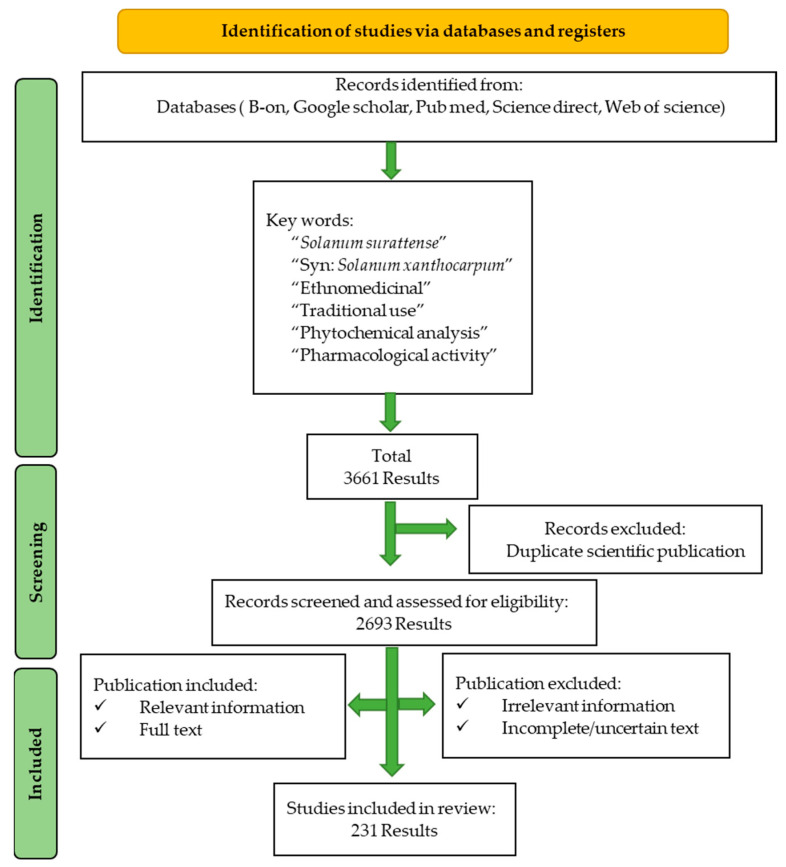
Screening of published data based on the PRISMA methodology.

The procedure to collect and select the data is depicted in Figure 2. From the initial 3661 scientific publications, after removing the duplicates and irrelevant and incomplete results, a total of 231 publications were selected and considered in this review.

### 2.2. Ethnomedicinal Uses

**Figure 3:**
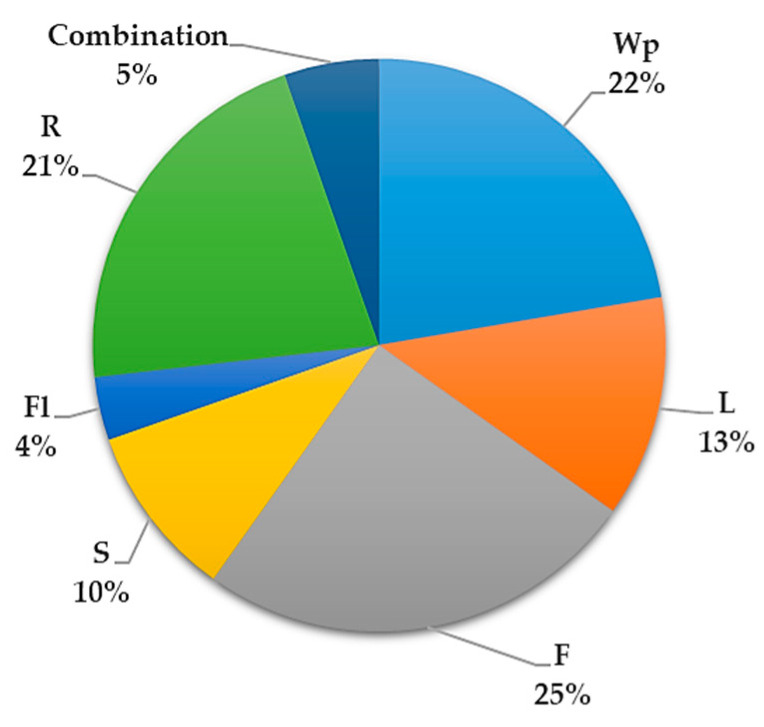
Ethnomedicinal uses of different parts of the *S. surattense* plant. Abbreviation: Wp—whole plant; L—leaf; F—fruit; S—seed; Fl—flower; R—root.

Different parts of *S. surattense* are utilized in traditional medicine, with the fruit (25%) being the most used part, followed by the whole plant (22%), root (21%), leaf (13%), seed (10%), plant parts mixture (combination) (5%), and flower (4%) (Figure 3). The predominant mode of usage is through decoction. A summary of the traditional uses of *S. surattense* in medicine is provided in Table S1 of the Supplementary Material.

The use of *Solanum surattense* in traditional medicine spans seven countries, with India and Pakistan being the primary users. Various parts of this plant are employed to treat multiple ailments [19,20,21].

Numerous reports [22,23,24,25,26,27,28,29,30,31,32,33,34,35,36,37,38,39,40,41,42] document the use of the whole plant in India, Iran, Nepal, and Pakistan. It is administered in various forms such as boiled, decoction, juice, paste, powder, topical application, cooked vegetable, and oral administration to address conditions like abdominal pain, arthritis, asthma, cough, colic pain, chronic constipation, fever, hemorrhoids, headache, inflammation, jaundice, leprosy, menstrual problems, paleness, skin issues, stomachache, throat diseases, vaginal infection, and urinary tract problems.

Several studies [43,44,45,46,47,48,49,50,51,52,53,54,55,56,57,58] highlight the medicinal use of *S. surattense* leaf in China, India, Pakistan, and Sri Lanka. These include topical paste for alopecia, oral infusion and smoke inhalation for asthma and cough, decoction for colds and dental issues, powder with jaggery for genital prolapse, marination with hot mustard oil or juice with *Piper nigrum* seed powder for joint pain, juice with black pepper and honey for respiratory diseases, and tincture and decoction for respiratory and urinary disorders.

In India and Pakistan, the flowers are commonly fried or powdered and mixed with honey to relieve asthma and cough. Additionally, a paste made from flowers and egg white is used in massages to alleviate arthritis [30,46,59,60].

The fruit is a significant part of the plant used in traditional medicine across Bangladesh, China, India, Nepal, and Pakistan [21,27,36,45,49,51,52,53,55,56,57,60,61,62,63,64,65,66,67,68,69,70,71,72,73,74,75,76,77,78,79,80]. It is used in various forms such as oral maceration for tumors and swelling, dried chewed fruit for toothache, topical paste for skin lesions, oral juice for diabetes and sore throat, ear drops for earache, decoction for joint pain and respiratory issues, and paste mixed with oil for massage to treat fever and paralysis.

Several articles [33,36,49,55,67,75,78,81,82,83,84,85] describe the use of seeds to treat amenorrhea, cardiac disease, dysmenorrhea, gastrointestinal problems, malaria, migraine, obesity, stomach pain, and toothache in forms such as decoction, rinsing solution, paste, vapor, and powder. In Pakistan, a decoction of the stem with black pepper and salt is orally administered for indigestion, fever, cough, and asthma [86]. Additionally, the root is traditionally used for a wide range of ailments, including abdominal pain, arthritis, asthma, cough, diabetes, fever, headache, hemorrhoids, inflammatory diseases, intestinal infection, jaundice, kidney problems, leprosy, measles, menstrual disorders, nervous system disorders, pain, phlegmatic cough, smallpox, snake bite, toothache, urinary troubles, and weakness through decoction, powder, smoke or fumigation, juice, paste, or tablet [87,88,89,90,91,92,93,94,95,96,97,98,99,100,101]. 

This species is also used in admixtures with other medicinal plants as polyherbal formulations (Pf) like the nine polyherbal formulations mentioned in Table 2. In Pf1, 4 g of mixed powder is given twice a day with water to treat urinary tract problems; in Pf2, 4 g (one teaspoonful) of mixed powder is given twice a day (morning and bedtime) with water for treating asthma/bronchitis; in Pf3, 4 g of mixed powder is given twice daily (morning and evening, 1 h before meals) with ginger juice for arthritis and rheumatic problems; in Pf4, 3 g of mixed powder is given twice daily (morning and at night before going to bed) with lukewarm water mixed with honey to cure colds; and in Pf5, 4 gm of mixed powder is given twice daily, morning and at bedtime with honey to treat throat diseases [24]. In Pf6, 10 mL of this mixture is given thrice a day for 20–30 days used for cough, fever, jaundice, bronchitis, and diabetes [91]. In Pf7, a mixture of *S. surattense* root (½ kg) and *Saccharum bengalense* root (½ kg) were orally administered (decoction) for 8–10 days for intestinal worm problems [71]. In Pf8, a paste of *S. surattense* root with black pepper (10 g) and ajwain (10 g) is given once a day for 3 days to decrease fever [98]. In the Indian pharmacopeia, Dasamula (Pf9) is used for different ailments like arthritis, asthma, Parkinson’s disease, gout, backache, anti-inflammatory, antioxidant properties, painful, inflammatory musculoskeletal disorders like osteoarthritis, and rheumatoid arthritis [102]. In another study, it was mentioned that an equal amount of *S. surattense* leaf and flower with *Leucas linifolia* leaf and flower are ground together, warmed, and applied to the swellings of joints for quick recovery [103].

### 2.3. Phytochemistry

Many researchers studied and published information on the chemical constituents of *S. surattense* in their scientific reports. In the methanol and ethanol extracts of *S. surattense* whole plant, the presence of steroidal alkaloids, steroidal saponins, methyl esters, phenolic acids, and fatty acids was observed [104,105].

**Figure 4:**
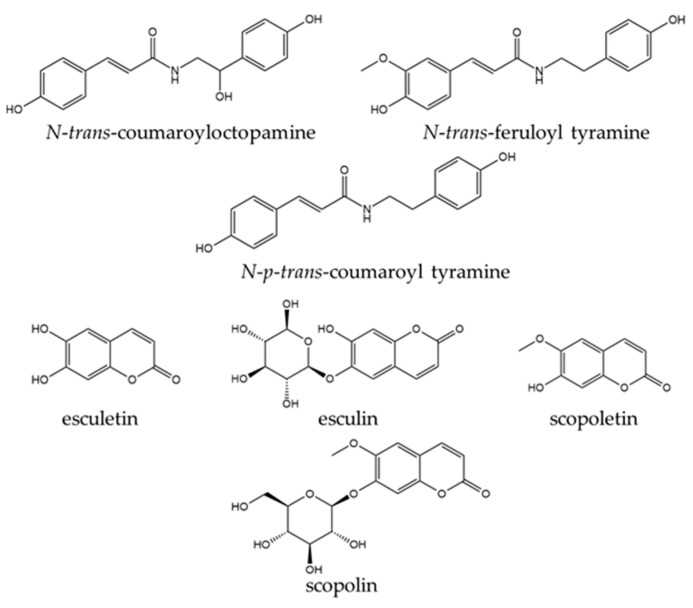
Examples of phenolic compounds isolated from different parts of *S. surattense*.

**Figure 5:**
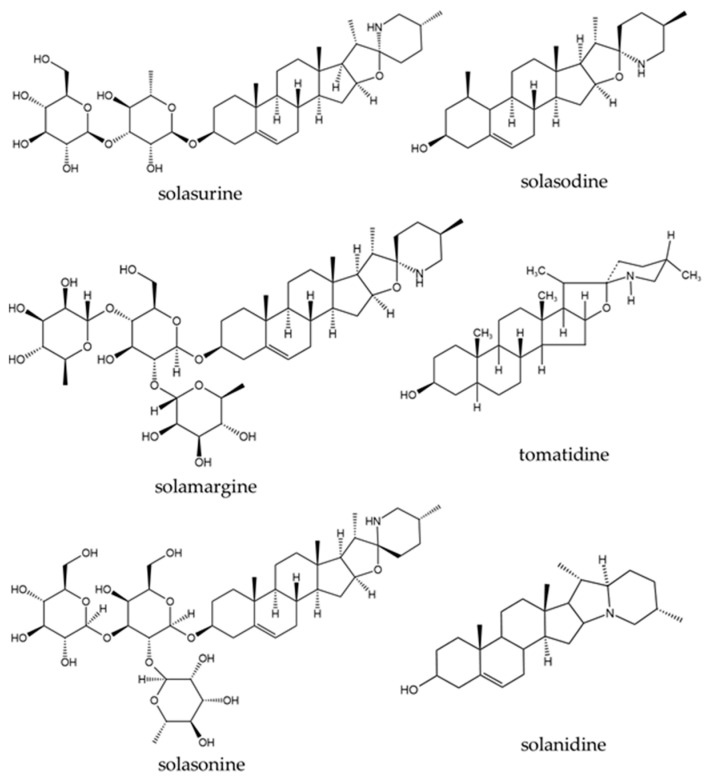
Examples of steroidal alkaloids isolated from different parts of *S. surattense*.

**Figure 6:**
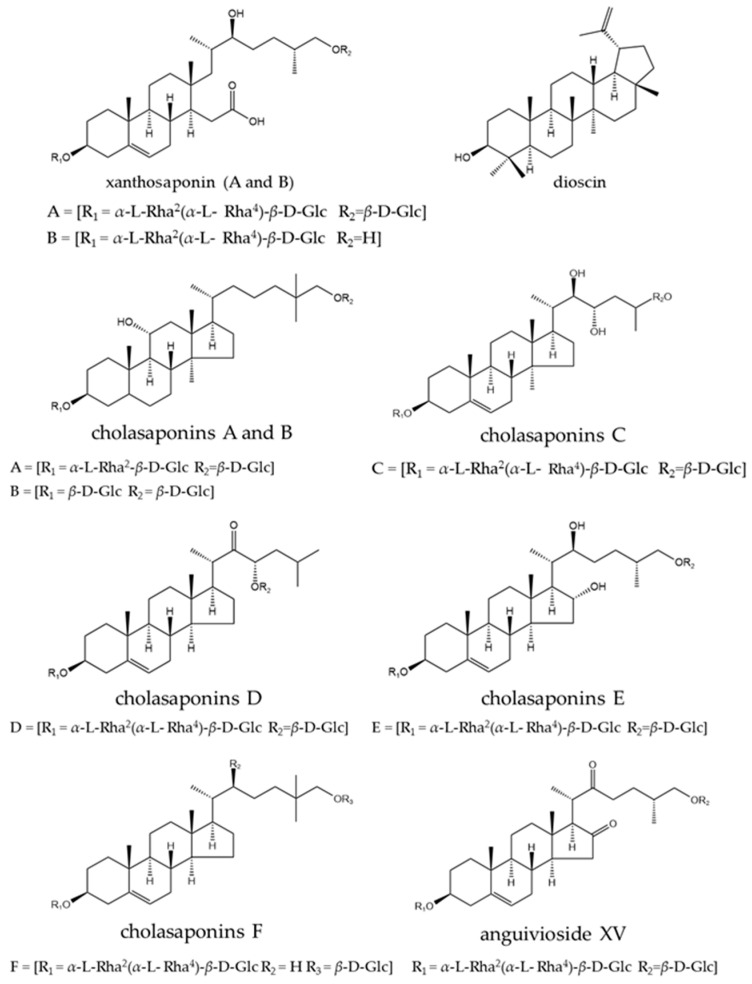
Triterpenoids isolated from different parts of *S. surattense*.

**Figure 7:**
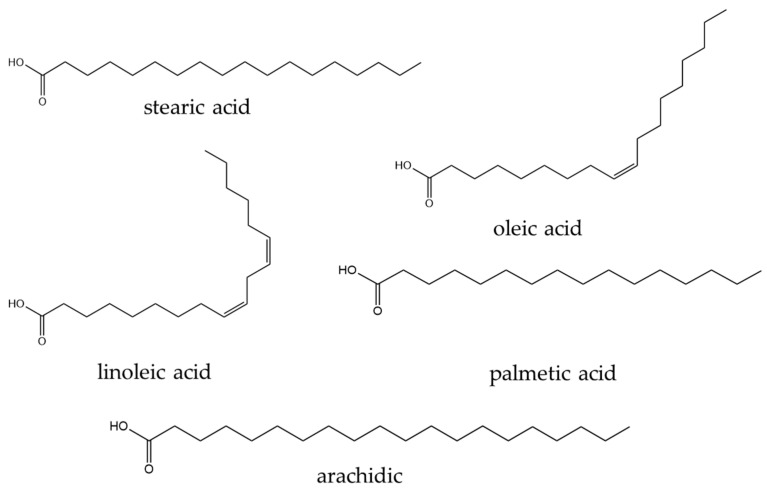
Some fatty acids isolated from different parts of *S. surattense*.

A total of 338 phytochemical constituents of various chemical classes were isolated from *S. surattense* [104,105,106,107,108,109,110,111,112,113,114,115,116,117,118,119,120,121,122,123,124,125,126,127,128,129,130,131,132,133,134,135,136,137,138,139,140,141,142]. The representative examples of the main compounds are presented in Figure 4, Figure 5, Figure 6 and Figure 7 and the total identified compounds are represented in Table S2 of the Supplementary Material.

Phenolics: 56 phenolic compounds (16.56%), including phenolic amides (1–8), phenolic acids (9–18), phenolic aldehydes (19), phenolic glycosides (20–22), flavonoids (23–39), coumarins (40–43), anthraquinones (44), lignans (45–55), and tannins (56) were found. These compounds were isolated from different parts of *S. surattense* (e.g., leaf, stem, fruit, and root) using polar solvents such as water, ethanol, methanol, and hydroethanolic mixtures [104,106,107,108,109,110,111,112,113,114,115,116,117,118,119]:

- Phenolic amides such as *N*-*trans*-feruloyl tyramine (1) and *N*-*p*-*trans*-coumaroyl tyramine (2) were identified in the whole plant of *S. surattense*. The compound 2-propenamide, *N*-[2-(dimethylamino)ethyl]-(3) was identified from the absolute alcohol extract of *S. surattense* leaf. Additionally, compounds such as dihydro-*N*-feruloyltyramine, *N*-*trans*-coumaroyltyramine, *N*-*trans*-coumaroyloctopamine, *N*-[2-(3,4-dihydroxyphenyl)-2-hydroxyethyl]-3-(4-ethoxyphenyl)-prop-2-enamide, and 3-(4-hydroxy)-*N*-[2-(3-methoxyphenyl-4-hydroxyphenyl)-2-hydroxy] (4–8) were identified from ethanolic extracts of the fruit part [7,8,12];
- Phenolic acids such as ferulic acid (9) were found in the methanolic extract of the whole plant [104], and evofolin B (10) was also recorded from the same plant part [106]. Chlorogenic acid (11) was isolated from methanolic extracts of the leaf, fruit, stem bark, and root [110,111]. Caffeic acid (12) was extracted from the methanol extract of aerial parts [112]. Compounds such as (1*R*,3*R*,4*R*,5*R*)-(-)-quinic acid (13) and 2-octylcyclopropene-1-heptanol (14) were recorded from ethanol and methanol extracts of the leaf [107,113]. Eugenol (15) was recorded from hydro-distilled oil extracts of the leaf and fruit, while methyl eugenol (16) and (*E*)-isoeugenol (17) were identified only from the fruit [114]. Butanedioic acid (18) was found in the ethanolic extract of the fruit part [115];
- Vanillin (19), a phenolic aldehyde, was identified in the ethanolic extract of *S. surattense* leaf, stem, and fruit, though it was found in significant amounts in the root. Some phenolic glycosides, including chlorogenic acid ethyl ester-4′-*O*-*β*-*D*-glucopyranoside (20), chlorogenic acid methyl ester-4′-*O*-*β*-*D*-glucopyranoside (21), and *p*-hydroxyphenyl acetonitrile-*O*-(6′-*O*-acetyl)-*β*-*D*-glucopyranoside (22), were identified from the ethanolic extract of *S. surattense* fruit [109];
- The flavonoid apigenin (23) was isolated from the methanolic extract of various plant parts, including leaf, fruit, petals, stem, and root. Other compounds such as isoquercitrin (24), gallocatechin (25), catechin (26), quercetin (27), flavone (28), luteolin (29), 4H-1-benzopyran-4-one, 5,7-dihydroxy-2-(4-hydroxyphenyl)-3-methoxy-(30), 5,7,4′-trihydroxy-8-methoxyflavone (31), 5,7,4′-trihydroxy-6-methoxyflavone (32), 5-hydroxy-8-methoxy-6,7-methylenedioxyflavone (33), 7-hydroxy-6-methoxycoumarin (34), fraxetin (35), 5-hydroxy-6,7,3′,4′-tetramethoxyflavone (36), 5-hydroxy-4′,6,7-trimethoxyflavone (37), and 5,3′-dihydroxy-6,7,4′-trimethoxyflavone (38) were identified from fruit parts using different solvents such as methanol (70%, 50%, and 30%), ethanol (95%), and aqueous ethanolic solutions [108,115,117,118]. Acetovanillone (39) was found only in the ethanolic extract of the fruit [115];
- Coumarins, including scopoline (40), scopoletin (41), esculin (42), and esculetin (43), were identified from petroleum ether and chloroform extracts of the leaf, fruit, and root parts [119]. The anthraquinone emodin (44) was extracted from the 50% ethanolic extract of the leaf, stem, and root parts;
- The lignans, including *threo*-1-(4-hydroxy-3-methoxyphenyl)-2-{4-[(*E*)-3-hydroxy-1-propenyl]-2-methoxyphenoxy}-1,3-propanediol (45), syringaresinol (46), coniferol (47), simulanol (48), balanophonin (49), glycosmisic acid (50), and tribulusamide A (51), were isolated from the whole plant of *S. surattense* [106]. Additionally, other lignans such as (7*R*,8*S*)-*threo*-glehlinoside C (52), 2*Z*-(7*S*,8*R*)-aegineoside (53), (7*R*,8*R*)-3,5-dimethoxy-8′-carboxy-7′-en-3′,8-epoxy-7,4′-oxyneolignan-4,9-diol (54), and glycerol *α*-guiacyl ether (55) were identified from the ethanolic extract of the fruit [109,115]. Only one tannin compound, quinic acid (56), was characterized from the ethanolic extract of the fruit [115].

A total of 12.50% of phenolic compounds were found to be biologically active. Among them, 3-(4-hydroxy)-*N*-[2-(3-methoxyphenyl-4-hydroxyphenyl)-2-hydroxy] (8), *p*-hydroxyphenyl acetonitrile-*O*-(6′-*O*-acetyl)-*β*-*D*-glucopyranoside (22), (7*R*,8*S*)-*threo*-glehlinoside C (52), 2*Z*-(7*S*,8*R*)-aegineoside (53), and (7*R*,8*R*)-3,5-dimethoxy-8′-carboxy-7′-en-3′,8-epoxy-7,4′-oxyneolignan-4,9-diol (54) showed significant anti-inflammatory activity in vitro. Additionally, caffeic acid (11) and tribulusamide A (51) demonstrated neuroprotective activity in vivo and hepatoprotective activity in vitro, respectively.

Alkaloids: Twenty-one alkaloid compounds (6.21%) were identified, including quinoline alkaloids and steroidal alkaloids [104,105,110,115,120,121,122,123,124,125,126,127,128,129,130,131].

- Quinoline alkaloids, isoquinoline (57) was isolated from the ethanolic extract of *S. surattense* fruit [115].
- Twenty steroidal alkaloids (58–77) have been reported from *S. surattense* [104,105,110,120,121,122,123,124,125,126,127,128,129,130,131]. Among them, compounds (58–64) were isolated from the methanolic extract of whole plant parts [104,120]. Five compounds (63–64, 66–68) were found in aerial parts using different solvents such as methanol, ethanol, petroleum ether, and chloroform. Ten compounds (63, 65, 67, 71–77) were characterized from the fruit parts, and two compounds (69–70) were isolated from the alcoholic extract of seeds. Notably, compounds 63, 64, and 67 were commonly found in the whole plant, aerial parts, fruit, and shoot [104,105,110,121,122,123,124,125,126]. Seven compounds (71–77) were characterized from *S. surattense* fruit extract using different solvents, including ethanol, and petroleum ether [127,128,129,130,131].

Among all the plant parts of the *S. surattense*, the fruit contains the most diverse secondary metabolites, particularly glycoalkaloids and steroidal alkaloids. In this study, steroidal alkaloids such as solamargine (63), solasodine (65), solasonine (67), solanidine (72), solasurine (74), tomatidine (68), and solanearpidine (77) were found as principal compounds in the fruit and throughout the whole plant [104,105,110,121,125,128].

23.81% of the alkaloids, including solamargine (63), khasianine (64), and (22*R*, 25*R*)-16*β*-*H*-22*α*-*N*-spirosol-3*β*-ol-5-ene3-*O*-*α*-*L*-rhamnopyranosyl-(1 → 2)-[*α*-L-rhamnopyranosyl-(1 → 4)]-*β*-*D*-glucopyranoside (66), exhibited anti-tumoral activity. Additionally, solasodine (65) and solasonine (67) demonstrated both anti-tumoral activity in vitro and antiurolithiatic activity in vivo.

Terpenoids: Terpenoids (78–214) are the major class, with 137 compounds (40.23%) identified. This class includes monoterpenoids, sesquiterpenoids, diterpenoids, and triterpenoids [104,107,111,112,114,115,121,122,127,131,132,135,138,139].

- Eight monoterpenoids (78–85), including 7*Z*-roseoside (78), linalool (79), camphor (80), *α*-terpineol (81), geraniol (82), isobornyl acetate (83), (*E*)-*β*-ionone (84), and dihydroactinidiolide (85), have been isolated from *S. surattense* leaf, fruit, seed, and root extracts using solvents like ethanol and aqueous solutions [105,132].
- Sixty-one sesquiterpenoids (86–146) have been identified from aqueous and methanol extracts of the leaf, fruit, seed, and root [113,114,132].
- Five diterpenoids (147–151), such as phytol (147), neophytadiene (148), (*E*,*E*)-geranyllinalool (149), lycopene (150), and carotenoids (151), have been characterized from aqueous, ethanol, and methanol extracts of the leaf and fruit [107,113,114,115].
- Sixty-three triterpenoids (152–214) have been identified from various parts, including the aerial parts, leaf, fruit, seed, stem, and whole plant, with the fruit being the major source [104,105,107,111,112,115,121,122,126,127,131,132,133,134,135,136,137,138,139].

A total of 18.25% of terpenoids, predominantly triterpenoids, exhibited various biological effects. These include dioscin, (22*R*, 23*S*, 25*R*)-3*β*, 6*α*, 23-trihydroxy-5*α*-spirostane 6-*O*-*β*-dxylopyranosyl-(1 → 3)-*O*-*β*-*D*-quinovopyranoside, (22*R*, 23*S*, 25*S*)-3*β*, 6*α*, 23-trihydroxy-5*α*-spirostane 6-*O*-*β*-*D*-xylopyranosyl-(1 → 3)-*O*-*β*-*D*-quinovopyranoside, (22*R*, 23*R*, 25*S*)-3*β*, 6*α*, 23-trihydroxy-5*α*-spirostane 6-*O*-*β*-*D*-xylopyranosyl-(1 → 3)-*O*-*β*-*D*-quinovopyranoside (153–156), solasaponin A–H (161–168), diosgenin (173), xanthosaponin A–B (174–175), cholesaponin A–F (203–208), and (22*S*)-25[(*β*-*D*-glucopyranosyl)oxy]-22-hydroxycholest-5-en-3*β*-yl-*O*-*α*-*L*-rhamnopyranosyl-(1 → 2)-*O*-[*α*-*L*-rhamnopyranosyl-(1 → 4)]-*β*-*D*-glucopyranoside (209). These compounds exhibited anti-tumoral activity, while carpesterol (177) and oleanolic acid (186) showed anti-diabetic activity in vitro and neuroprotective activity in vivo, respectively.

*β*-Sitosterol (182) and diosgenin (173) were obtained from the hydroethanolic extract of *S. surattense* calli. A higher content of *β*-sitosterol and diosgenin was quantified in tissue culture than in the fully grown *S. surattense* plant [126]. Gupta and Dutt investigated the constituents of the semi-drying oil obtained from the benzene extract of *S. surattense* seed and reported the presence of fatty acids, including oleic acid, linoleic acid, palmitic acid, stearic acid, arachidic acid, and unsaponifiable matter (a mixture of two sterols) [141].

Lipids: Fifty-one compounds (15.08%) identified as lipids (215–266) encompass fatty acids, aldehydes, fatty alcohols, fatty amides, sphingolipids, oxylipins, and phenolic lipids [104,106,107,113,114,115,140,141,142].

- Eighteen fatty acids (215–232) have been isolated from the leaf, stem, fruit, root, and whole plant of *S. surattense*, with most of the compounds found in the leaf [104,107,113,114,115,140,141].
- Eleven aldehydes, including nonanal (233), (2*E*,4*E*)-decadienal (234), dodecanal (235), tridecanal (236), tetradecanal (237), pentadecanal (238), hexadecanal (239), 9,12,15-octadecatrienal (240), tetracosanal (241), pentacosanal (242), and hexacosanal (243), have been isolated from aqueous extracts of *S. surattense*, especially from the fruit [114].
- Three fatty alcohols, 1-octanol (244), (6*Z*)-nonenol (245), and (*Z*)-dihydroapofarnesol (246), were isolated from aqueous extracts of the fruit and seed, while two fatty amides, (9Z)-octadecenamide (247) and octadecanamide (248), were identified from the seed [114].
- Additionally, eight sphingolipids (249–256), seven oxylipins (257–263), and three phenolic lipids (264–266) have been identified from the fruit of *S. surattense* [142].

Lipids, accounting for 34.62%, were found to have anti-inflammatory potential in vitro. These include compounds such as 6′′-*O*-acetyl soya-cerebroside I (249), soya-cerebroside I-II (250–251), 2*S*,3*S*,4*R*,8*E*-2-(2′*R*-2′-hydroxyhexacosanosylamino)-octadecene-1,3,4-triol (252), gynuramide I-IV (253–256), methyl 9*S*,10*S*,11*R*-trihydroxy-12*Z*,15*Z*-octadecadienoate, 9*S*,10*S*,11*R*-trihydroxy-12*Z*,15*Z*-octadecadienoic acid, methyl 9*S*,10*S*,11*R*-trihydroxy-12*Z*-octadecenoate, 9*S*,10*S*,11*R*-trihydroxy-12(*Z*)-octadecenoic acid, methyl 9*S*,12*S*,13*S*-trihydroxyoctadeca-10*E*,15*Z*-dienoate, 9*S*,12*S*,13*S*-trihydroxy-10*E*-octadecenoate, 2′*S*-20-hydroxy arachidic acid glycerol ester (257–263), 2′*S*-20-*O*-caffeoyl-20-hydroxy arachidic acid glycerol ester, 2′*S*-22-*O*-caffeoyl-22-hydroxy-docosanoic acid glycerol ester, and 2′*S*-22-*O*-*p*-hydroxy-phenyl propionyloxy-22-hydroxy-docosanoic acid glycerol ester (264–266).

Some lipid compounds influence growth factors through phytohormones such as auxins (IAA and IBA), kinetin (Kn), and gibberellic acid (GA) in the callus culture of *S. surattense* [143,144]. Additionally, certain compounds of this chemical class exhibited anti-inflammatory activity in a paw edema carrageenan-induced inflammation model in rats and demonstrated in vitro anti-cancer activity against HeLa and U937 cell lines [125,145].

Several sources of the scientific literature have reported various quantitative analysis results in different plant parts like leaf, fruit, stem, stem bark, root, and root bark of *S. surattense* [113,146,147,148,149,150,151]. Among the different solvents used in extraction, methanol was the most used one for quantitative analysis. The quantitative analysis focused on determining the total phenolic, flavonoid, tannin, and terpenoid contents. The phenolic content was highest in the ethanol extract of *S. surattense* leaf, measuring 46.7 GAE/mg [146], followed by the acetone extract of *S. surattense* root at 28.9 g/100 g [150], while the lowest value of 4.975 GAE/mg was found in the methanol extracts of the fruit [147], indicating that ethanol solvent is probably the best solvent for more phenolic constituents. 

The flavonoid content was highest in the ethyl acetate and acetone extracts of *S. surattense* fruit, measuring 162.4 ± 0.15 μg QE/mg and 148 ± 0.18 μg QE/mg, respectively [151]. In contrast, the methanol extracts of *S. surattense* leaf showed the lowest value at 2.48 ± 0.6 Rutin/µg [148]. In addition, the acetone extract of *S. surattense* root has the highest total tannin content of 18.7 g/100 g extract [150]. Regarding total terpenoid content, the highest content was 6.3 ± 1.2 GAE/mg in the methanol extract of *S. surattense* root [113]. Moreover, the quantification analysis differs in results based on plant parts, extracting solvents, and way of result expression. More details are presented in Table 3.

### 2.4. Pharmacological Studies

**Figure 8:**
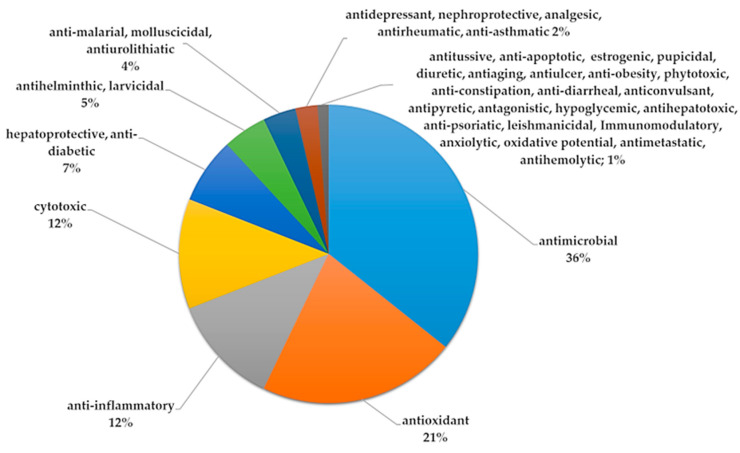
Different biological activities (%) of *S. surattense*.

**Figure 9:**
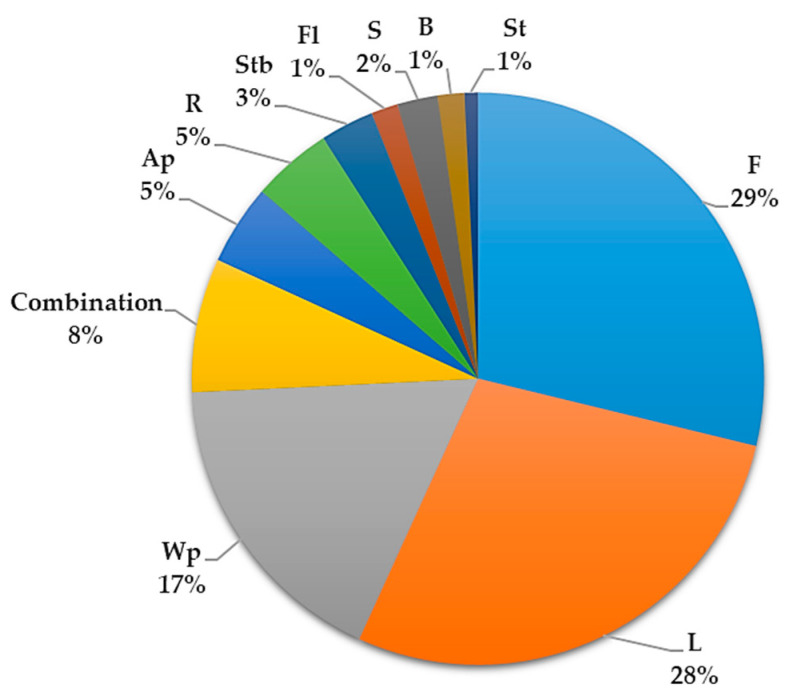
Percentage of different plant parts of *S. surattense* used in different biological activities. Abbreviation: F—fruit; L—leaf; Wp—whole plant; Ap—aerial part; R—root; Stb—stem bark; Fl—flower; S—seed; B—bark; St—stem.

Extracts from the whole plant and various parts of *Solanum surattense*—including aerial part, leaf, fruit, flower, seed, stem, stem bark, and root—have been extensively studied for their biological activities. These results are comprehensively summarized in Table 4 and illustrated in Figure 8 and Figure 9. Although there are no formal scientific reports on using *S. surattense* specifically against piles, traditional medicine in regions like India, Pakistan, and Bangladesh frequently employs this plant for such conditions, often lauding it as a remarkable therapeutic agent. Notably, the ancient Indian text “Materia Medica” references *S. surattense*, particularly its root, for treating a variety of ailments, including piles [152], using methods like fumigation [89].

#### 2.4.1. Anti-Inflammatory Activity

The anti-inflammatory properties of *S. surattense* are well-documented, particularly for its ethanolic extracts from leaf and fruit. These extracts exhibit significant activity in both in vivo and in vitro experiments [118,185,191,198]. The alcoholic extract of aerial parts, as well as a gel formulated with Carbopol 940 polymer, have been evaluated for wound healing potential using excision and incision wound models in male Wistar rats (180–220 g).

For the excision wound model, rats were divided into eleven groups (six animals per group):

- Groups 1 and 2: Normal topical control with Carbopol gel and normal oral control with distilled water;
- Groups 3 and 4: Diabetic topical control and diabetic oral control with Carbopol gel and distilled water, respectively;
- Groups 5 and 6: Diabetic treated topically with aloe vera cream and orally with aloe vera juice;
- Groups 7–10: Diabetic treated topically and orally with ethanolic extract of *S. surattense* ESX gel (5% *w*/*w* and 10% *w*/*w*) and ESX (100 mg/kg and 200 mg/kg), respectively;
- Group 11: Diabetic treated with both topical (ESX gel 10%) and oral (200 mg/kg) treatments.

Significant effects were observed across all doses for both topical and oral treatments. The combination treatment (ESX Gel 10% + ESX 200 mg/kg) showed the most substantial wound closure (93.50 ± 1.60%), followed by ESX Gel 10% (88.33 ± 2.24%) and ESX 200 mg/kg (85.16 ± 1.27%). For the incision wound model, animals were divided into nine groups (excluding Groups 10 and 11 from the excision wound model), demonstrating a significant increase in wound-breaking strength (WBS), particularly in those treated with the combination therapy [167].

Parmar et al. (2010) investigated the anti-inflammatory effects of ethanolic leaf extracts using Sprague-Dawley rats (140–160 g) in a carrageenan-induced paw edema model. The extract (50–400 mg/kg, p.o.) significantly inhibited paw swelling at doses of 100, 200, and 300 mg/kg, with edema inhibition percentages of 31.57%, 46.31%, and 45.26%, respectively, at 3 h. These results were highly significant (*p* < 0.01) compared to the control [190].

Aqueous extracts of dried *S. surattense* fruit were evaluated for anti-inflammatory activity using the carrageenan-induced paw edema assay in Wistar Albino rats (150–300 g). Six groups were categorized: positive control, negative control, *S. surattense* group, *Cassia fistula* group, combinations 1 and 2. The *S. surattense* dried fruit extract showed superior anti-inflammatory properties compared to *C. fistula*, with the highest effects at a 500 mg/kg dose. The combination of *S. surattense* and *C. fistula* (1:1) exhibited synergistic efficiency, achieving 75% inhibition compared to 81% for diclofenac sodium [204].

Key anti-inflammatory components from *S. surattense* include stigmasterol [234], carpesterol [235], and diosgenin [236]. Other compounds such as solanidine, α-solanine, and α-chaconine also possess significant therapeutic potential against inflammation. Chronic inflammation, often seen in autoimmune diseases, cancer, vascular disorders, and arthritis, can be addressed by targeting key molecular pathways. Lupeol, identified in *S. surattense*, demonstrates immense anti-inflammatory potential as a multi-target agent, affecting pathways such as NFκB, cFLIP, Fas, Kras, PI3K/Akt, and Wnt/β-catenin. Remarkably, lupeol at therapeutic doses shows no toxicity to normal cells, making it a promising candidate for both preventive and therapeutic applications against inflammation [237].

#### 2.4.2. Anti-Diabetic Activity

In the quest for advanced and effective anti-diabetic drugs, substantial research highlights the potential of various plants. *S. surattense* has shown prominent anti-diabetic properties comparable to the standard drug “Glibenclamide”. Sridevi et al. (2007) demonstrated that *S. surattense* leaf extract possesses antihyperglycemic potential in Streptozotocin-induced diabetic male Wistar Albino rats (150–300 g). Five groups (*n* = 6) were categorized: group I: normal rats receiving 2% gum acacia only; group II: normal + leaf extract (100 mg kg^−1^ bw) in 2% gum acacia; group III: diabetic control rats (STZ-40 mg kg^−1^ bw); group IV: diabetic + leaf extract (100 mg kg^−1^ bw) in 2% gum acacia; group V: diabetic + glibenclamide (600 μg kg^−1^ bw) in 2% gum acacia for this study. Extended oral administration of 100 mg/kg b.w. of leaf extract for 45 days significantly reduced blood glucose levels and increased insulin levels [177].

Gupta et al. (2011) identified *β*-sitosterol from *S. surattense* as having promising antidiabetic properties. For this study, male Wistar Albino rats (170–190 g) were used, and they were divided into nine experimental groups (*n* = 9), including a control group, a diabetic group, and BS- and glibenclamide-treated diabetic groups. A 21-day experiment showed increased serum insulin levels in the treated group compared to controls. Enhanced levels of pancreatic antioxidants, such as SOD, CAT, GSH, GST, GPx, and ascorbic acid, were also observed, confirming *β*-sitosterol’s antidiabetic and antioxidant effects [221].

#### 2.4.3. Anti-Tumor Activity

*S. surattense* has a wide range of pharmacological properties, including anticancer efficiency. Both polar and nonpolar solvent extracts of *S. surattense* leaf and fruit showed potential inhibitory activity against cancer cell proliferation [118,151,238]. The methanol extract of *S. surattense* whole plant was responsible for apoptosis-inducing activity and causes cell death [239]. In another study, *S. surattense* leaf extract with the nanoparticle solution (silver nanoparticle solution (AgNPs)) also showed significant cytotoxicity [181].

The presence of different secondary metabolites such as lupeol, apigenin, stigmasterol, solancarpine, carpesterol, solamargine, diosgenin, and steroidal alkaloids enhances the cytotoxic activity. Lupeol, apigenin, and solamargine exhibit the potentiality of antitumor activity by enhancing apoptosis. Lupeol possesses up-regulation of melanogenesis through activation of the p38 MAPK pathway against B16 2F2 melanoma cells. Solamargine induces non-selective cytotoxicity and P-glycoprotein inhibition. Again, the appearance in solamargine-treated cells of chromatin condensation, DNA fragmentation, and a sub-G1 peak in a DNA histogram suggests that solamargine induces cell death by apoptosis. Apigenin decreases the genotoxic damage induced by mitomycin C and cyclophosphamide and activates anti-cancerous potentials, thereby reducing the chances of developing secondary tumors [240,241,242,243]. Apigenin, quercetin, fisatin, and luteolin recorded from *S. surattense* fruit extracts act as potential inhibitors of cancer cell proliferation, e.g., human lung cancer cell lines (HOP-62) and leukemic (THP-1) cell lines [151,244]. Cham (2017) reported that solamargine and solasodine showed cytotoxicity against Hep 2 B cells of 10 µm [245]. Sethi et al. (2018) mentioned that diosgenin exhibited apoptosis activity on HCT 116 cell lines (human colon carcinoma cell lines) [168]. These findings of the study confirmed that the steroidal constituents (from *S. surattense*) are responsible for apoptosis-inducing activity and cause cell death. Through these systematic investigations, it is well explained how inducing apoptosis and cell death could potentially develop therapeutic drugs to overcome cancer. 

#### 2.4.4. Antioxidant Activity

Antioxidants are a very important factor in improving health problems, and they can be isolated from traditional medicinal plants. Antioxidants can protect against oxidative damage [169]. Some plants have rich resources of antioxidants which have potential effects against reactive oxygen/nitrogen species. Therefore, the determination of natural antioxidant compounds of plant extracts will be helpful for the development of new drug candidates for antioxidant therapy [246]. The leaf contains a higher quantity of phenols and flavonoids than the stem and fruit. The presence of phenols and flavonoids in *S. surattense* has become a natural source of potential antioxidants and can be used as a medicine against diseases caused by free radicals [147]. Leaf extract of *S. surattense* enhanced the level of antioxidant enzymes superoxide dismutase and glutathione peroxidase in alloxan-induced animal models [173]. Meena et al. (2010) mentioned that methanolic and ethanolic extracts of *S. surattense* have potential antioxidant properties [29]. Joseph et al. (2011) estimated higher levels of total phenolics (28.9 g/100 g extract) and tannins (18.7 g/100 g extract) in the acetone extract of *S. surattense* root that has the potentiality to exhibit higher activity against DPPH, ABTS^+^, OH^−^ radical scavenging, and phosphomolybdenum reduction [150]. Nithiyanantham et al. (2012) reported that *S. surattense* fruit aqueous extract exhibited good scavenging and reducing potentiality against DPPH and FRAP with the values of 2.1 ± 0.2 and 2.5 ± 0.0 (boiled) g extract/g DPPH, and 7.0 ± 0.0 and 28.5 ± 0.0 (boiled) µg extract/mmol Fe(II) [149]. Muruhan et al. (2013) reported that the significant scavenging efficiency of *S. surattense* leaf extracts against 2,2-diphenylpicrylhydrazyl has shown remarkable antioxidant activity at all test doses in a dose-dependent manner [146]. Kumar and Pandey (2014) measured free radical scavenging activity by DPPH assay at different concentrations of 250, 500, and 1000 μg/mL. In the DPPH radical scavenging assay, most of the *S. surattense* fruit extracts (chloroform, ethyl acetate, acetone, ethyl alcohol, and water extracts) demonstrated appreciable radical scavenging activity at a 250 μg/mL concentration, revealing considerable antioxidant potential in the extracts where total flavonoid contents (ranged between 10.22–162.49 μg quercetin equivalent/mg) showed a positive correlation with antioxidant activity [151]. Shah et al. (2013) explained that fruit extracts possess an appreciable amount of radical scavenging activity (about 80%) at a concentration of 250 µg/mL, but no changes at increased test dose concentrations of 500 and 1000 µg/mL (due to the saturation effect) [197]. Poongothai et al. (2011) reported that methanol extract of *S. surattense* leaf enhanced the level of antioxidant enzymes catalase (CAT), superoxide dismutase (SOD), and glutathione (GSH) peroxidase in alloxan-induced animal models. Antioxidants increased to the normal level, and the efficiency showed similar to standard drug glibenclamide. The antioxidant potentiality of *S. surattense* leaf extracts might be attributed to the presence of phenolic and flavonoid compounds in high quantities [173]. According to Azhar et al. (2020), the methanol extract of the stem bark, with an IC_50_ value of 0.323102 mg ml^−1^, had the highest potential for antioxidant activity [148]. These findings confirm that *S. surattense* would be an effective natural source of antioxidants.

#### 2.4.5. Antibacterial Activity

Many studies have highlighted the antibacterial properties of plant extracts from *S*. *surattense*. Ahmed et al. (2009) found that methanol and aqueous extracts from different plant parts (fruit, shoot, root) were effective against both gram-positive and gram-negative bacteria, with the fruit extract showing the highest activity [42]. Sheeba (2010) reported significant antibacterial effects of ethanol leaf extracts against eight bacterial strains [179]. Nithiyanantham et al. (2012) noted the antimicrobial activity of aqueous fruit extracts against gram-negative bacteria [149]. Abbas et al. (2014) observed that fruit extracts inhibited various bacterial strains, with varying zones of inhibition [195]. Mickymaray et al. (2016) found ethanolic aerial part extracts effective only against *E*. *coli* [247]. Another study showed high sensitivity of different plant part extracts to *Klebsiella pneumoniae* and *Salmonella typhi*, with varying effectiveness against other bacteria [228]. Choudhary et al. (2016) reported that the whole plant aqueous extract exhibited significant antibacterial activity at high concentrations [154]. Different solvent extracts of *S. surattense* leaf showed varying degrees of effectiveness against several bacterial strains, with methanol extracts being the most effective [113]. Another study demonstrated that methanol, ethanol, and chloroform extracts of the whole plant had different minimum inhibitory concentrations against *Staphylococcus cohnii*. Overall, these studies highlight the broad antibacterial potential of *S. surattense* extracts [153].

#### 2.4.6. Antiviral Activity

The anti-HIV reverse transcriptase (RT) activity of different solvent extracts of *S. surattense* fruit has been evaluated, showing dose-dependent inhibitory effects at concentrations of 0.6 and 6.0 μg/mL. Benzene and acetone extracts demonstrated significant RT inhibition [151]. 

#### 2.4.7. Antifungal Activity

Methanol extracts of *S. surattense* fruit have shown broad-spectrum antifungal effectiveness, inhibiting the growth of fungal strains such as *Trichoderma viride*, *Aspergillus niger*, *A. flavus* and *A. Fumigatas*. Singh et al. (2007) reported that the antifungal efficiency of isolated steroidal glycosides exhibited inhibitory effects on the radial growth of *A. niger* and *T. viride*, where *T. viride* exhibited the highest susceptibility and showed the highest growth inhibition antifungal effect of plant extracts compared with the standard drug amphotericin-B [196]. David et al. (2010) reported that among the aqueous, ethanolic, and methanolic extracts of *S. surattense* seed, ethanol seed extracts showed high antifungal activity against *Candida albicans*, *C. tropicalis*, *A. niger*, *A. fumigates* and *A. flavus*; methanol extracts showed activity against *A. fumigatus* and *Rhizopus oryzae*; and aqueous extracts showed activity only against *C. albicans* [218]. Jinal and Amaresan (2020) also evaluated that aqueous-ethanol root extract showed antifungal activity against *R. solanacearum* and *F. oxysporum*, respectively [226]. 

#### 2.4.8. Antihelminthic Activity

Aqueous and ethanolic extracts of *S. surattense* fruit showed anthelminthic activity against *Pheritima posthuma*, whereas aqueous extract showed better anthelmintic activity in comparison to the ethanol extract [200]. Priya et al. (2010) recorded that aqueous, hydroethanolic, and ethanolic extracts of *S. surattense* showed anthelminthic activity at 25, 50, and 100 mg/mL conc. Here, ethanolic extracts showed a remarkable anthelmintic potentiality compared to aqueous and hydroethanolic extracts at a concentration of 100 µg/mL [159]. In another study, ethanol extract of *S. surattense* fruit showed anthelminthic activity against *P. posthuma* at different concentrations (10, 25, and 50 mg/mL) and caused paralysis and death [199]. Barik et al. (2018) mentioned similar findings on the anthelminthic efficiency of ethanolic and aqueous extracts of *S. surattense* fruit [205]. 

#### 2.4.9. Cardiovascular Activity

Pasnani (1988) investigated the cardiovascular effects of Abana, a formulation containing solasodine from *S. surattense*. The study found that Abana caused direct sensitization of the atrium and downregulation of beta-adrenoceptors [248].

#### 2.4.10. Hepatoprotective Activity

Hepatic diseases, often caused by oxidative stress and inflammation, are a serious health concern. Traditional Ayurvedic medicine uses *S. surattense* fruit to treat liver disorders. Gupta et al. (2011) investigated the hepatoprotective potential of *S. surattense* fruit ethanolic extract applied to CCl4-induced (carbon tetrachloride) acute liver toxic experimental animals. A total of six groups (*n* = 6) of Sprague-Dawley rats were made, where Group I was considered as the control group and administered a single daily dose of carboxymethyl cellulose (1 mL of 1%, *w*/*v*, p.o. body weight). Group II received carbon tetrachloride (1 mL/kg b.w., i.p. 1:1 *v/v* mixture of CCI4 and liquid paraffin) alone, while groups III, IV and V received orally 100, 200 and 400 mg/kg body weight of ethanolic (50%) extract of *S. surattense* in (1%, *w*/*v*, CMC), respectively. And group VI received silymarin, the known hepatoprotective compound (standard), at a dose of 100 mg/kg, p.o., along with carbon tetrachloride. The fruit extract at a dose of 400 mg/kg showed a significant effect on lowering the serum marker enzymes, where reduction in the level of serum marker enzymes is comparable between the CCl4 group and Silymarin. The percentage protection in marker enzyme of treated groups (III, IV, and V) at the dose of 400 mg/kg was AST 67.71, ALT 75.66, ALP 54.52 compared to the silymarin treated group VI at the dose of 100 mg/kg as AST 70.36, ALT 77.40, ALP 59.80 [221].

Ghassam et al. (2014) conducted a similar study, finding that the leaf methanolic extract decreased serum LDH, ALP, and AST levels significantly (1.7-fold, 1.6-fold, and 1.8-fold, respectively). At a dose of 200 mg/kg extract, SOD, CAT, GST, and GSH levels in liver homogenates were increased (1.78 ± 0.13, 34.63 ± 1.98, 231.64 ± 14.28, 8.23 ± 0.48). Albino Wistar rats weighing 180–200 g were used in this study. Animals were categorized into five groups (*n* = 6); group I: sterile distilled water (positive control), group II: CCl4 (negative control), group III: 100 mg/kg b.w. of SXAF (*S. surattense* active fraction) orally for 14 d + single oral dose of CCl4 on the 15th day (1 mL/kg b.w.), group IV: 200 mg/kg b.w. of SXAF orally for 14 d + single oral dose of CCl4 on the 14th day and group V: 25 mg/kg b.w. of silymarin orally for 14 d + single oral dose of CCl4 on the 14th day. Histopathological examination also showed lowered liver damage in CCl4-induced groups [183].

Singh et al. (2016) noticed the hepatoprotective potential of *S. surattense* fruit extracts combined with *Juniperus communis* against Paracetamol (PCM) and Azithromycin (AZM) induced hepatic injury. Wistar albino rats of either sex (150–200 g) were used, and total experimental procedures were conducted in eight experimental groups (*n* = 6) in this study. The administration of AZM and PCM significantly produced liver toxicity by increasing the serum level of hepatic enzymes (SGPT, SGOT, and ALP) and oxidative parameters causing liver damage in rats. Administration of *S. surattense* fruit extract (200 and 400 mg/kg) and *J. communis* (200 and 400 mg/kg) for 14 days significantly attenuated the liver enzymes (SGPT, SGOT, and ALP) in AZM and PCM-treated animals. The findings indicated that *S. surattense* fruit extracts have hepatoprotective potential due to their synergistic antioxidant properties [193].

Jigrine is a combination of 14 medicinal plants including *S. surattense* in aqueous extract form, used to treat various liver conditions as a polypharmaceutical herbal hepatoprotective formulation. Najmi et al. (2005) investigated the DPPH-free radical scavenging activity, hepatoprotective, and antioxidant activity of Jigrine against galactosamine-induced hepatotoxicity in rats. Wistar strain albino rats (150–200 g) were used for the study and categorized into four groups (*n* = 6). Both positive and negative controls were used here. In Galactosamine-induced animals, a significant increase in serum AST, ALT, urea, and tissue TBARS levels was observed and analyzed to assess liver function. Administration of Jigrine (1 mL/kg, p.o.) for 21 days decreased the levels of the above indices significantly. The biochemical data exhibited significant hepatoprotective activity against galactosamine-induced rat models [249]. 

According to this review, antimicrobial (36%), antioxidant (21%), anti-inflammatory (12%), and cytotoxic activities (12%) were the most reported activities of this species, where antimicrobial activity showed the highest percentage (Figure 8).

Similarly, the most used extracts were ethanolic (47.37%), and the most used plant parts were fruit (29%) and leaf (28%) (Figure 9).

### 2.5. Pharmacological Activity of Secondary Metabolites of S. surattense

Fifty-five isolated compounds from *S. surattense* that were biologically tested and studied for their potential bioactivities by different researchers demonstrated anti-tumor and anti-inflammatory are the most remarkable activities (Table 5).

Several active compounds from *S. surattense* have been reported to strongly bind to the target proteins, potentially inhibiting their functions or mechanisms, indicating their therapeutic potential by in silico method. Marker secondary metabolites such as quercetin and heptahydroxy flavone interact with the following proteins: AKT1 (AKT Serine/Threonine Kinase 1), BCL2L1(Bcl-2-like protein 1), EGFR (Epidermal Growth Factor Receptor), ESR (Estrogen Receptor), H1F1-α (Hypoxia-inducible Factor 1-alpha), HRAS (Harvey Rat Sarcoma Virus), mTOR (mammalian target of rapamycin), TNF (Tumor Necrosis Factor). Also, solanidine interacts with AKT1, EGFR, H1F1-α, mTOR; esculetin, with BCL2L1 and TNF; Leptinidine, with ESR; and Verazine with HRAS and creates a strong binding force with these target proteins. These interactions suggest that these compounds can inhibit cell proliferation and have shown therapeutic effects against hepatocellular carcinoma [250]. 

In another study, Hasan et al. (2020) reported that the C3-like protease of SARS-CoV-2 raises the possibility of their acting therapeutically against the virus. Marker secondary metabolites from *S. surattense*, such as *α*-solamargine, bind strongly with both catalytic residues His41 and Cys145, and other residues including Ser46, Ser144, His163, Asn142, Glu166, Met49, and Gln189. It also binds with inhibitor N3 residues: His41, Met49, Phe140, Leu141, Asn142, Gly143, His163, His164, Glu166, Leu167, Pro168, Gln189, Thr190, and Ala191. Similarly, solanine interacts with catalytic residues Cys145 and His41 as well as other residues such as His163, His164, Met165, and Pro168 and Asp187, Gln189, and Ala191, and interacts with inhibitor N3 residues Met49, His163 and His164. Solasurine interacts with Phe8, Pro9, Ile152, Tyr154, Pro293, Phe294, Val297, and Arg298; tomatidenol binds with catalytic residue Cys145, and other residues like Ser144, Pro168 and Ala191. Carpesterol interacts with Arg40, Cys85, Phe 134, and Pro 184, creating a strong bond [251]. 

Also, apigenin, chlorogenic acid, stigmasterol, and stigmasterol glycoside were found to create strong bonds with target proteins such as EGFR, TP53, ERBB2, and STAT3, providing therapeutic effects against psoriasis [252]. 

### 2.6. Clinical Studies

Govindan et al. (1999) performed a pilot experiment on the clinical efficacy and safety of a single dose in mild to moderate bronchial asthma resulting in relief from asthmatic symptoms after 1 h, and its effect lasted for about 6–8 h. The respiratory functions (FVC, FEV1, PEFR, and FEF25–75%) were assessed by using a spirometer before and 2 h after the oral administration of 300 mg powder of the whole plant *S. surattense*. Treatment with *S. surattense* significantly improved the various parameters of pulmonary function in asthmatic subjects [26]. Govindan et al. (2004) studied the clinical efficacy of *S. surattense* and *S. trilobatumin* in bronchial asthma. For the clinical efficacy, a dose of 300 mg for 3 days was administered orally in mild to moderate bronchial asthma. *S. surattense* and *S. trilobatum* produced a progressive improvement in the ventilatory function of asthmatic individuals over 3 days. The scores for rhonchi, cough, breathlessness, and sputum decreased with these drug treatments. The improvement in PEFR and the reduction in other symptom scores clearly indicate a bronchodilator effect and a decrease in edema and secretions in the airway lumen. These clinical trials proved the anti-asthmatic potential, which is important for the management of asthma [253]. Another experiment was performed by Divya et al. (2013) where anti-asthmatic activity of the polyherbal ayurvedic drug was observed in in vitro and in vivo conditions. This trial on 60 bronchial asthmatic patients resulted in significant improvement in pulmonary expiratory flow rate (PEFR), forced vital capacity (FVC), and forced expiratory volume (FEV), indicating that constant improvement was observed throughout the follow-up with no recurrence of bronchial constriction [254]. Joshi et al. (2021) conducted a randomized clinical trial on gingivitis on 75 patients considering the safety and efficacy to assess the properties of two herbal mouth rinses (*S. surattense* and *Acacia catechu* Willd) and compare the herbal mouth rinses with Chlorhexidine (Gold standard) [255].

Especially in India, several herbal medicines based on *S. surattense* are available on the market, such as *S. xanthocarpum* powder and tablets (Bharat Herbal, India), Indukantham Kashayam (Planet Ayurveda, Punjab, India), Koflet (Himalaya Wellness Company, Bengaluru, India), Mother Tincture *Solanum xanthocarpum* (Dr. Willmar Schwabe India Pvt. Ltd., Delhi, India), and Kantakari powder and capsules (DR WAKDE’S Natural Health Care, London, UK). However, these products are neither approved nor controlled by the Indian Drug Control Agency, and no information regarding their quality, safety, and efficacy is available in the literature. Additionally, a Chinese patent was identified, describing the preparation and administration of an ethanolic herbal formulation from *S. surattense* fruit. This formulation contains 80 to 99% total weight (wt%) of solancarpine (30–50 wt%), solamargine (10–30 wt%), and solasurine (30–50 wt%), and is intended for the prevention and treatment of diseases such as tumors, diabetes, asthma, and coronary heart disease [256].

### 2.7. Toxicological Studies

Baskar et al. (2018) studied the toxic effect on *Helicoverpa armigera* (Hub.), *Culex quinquefasciatus* (Say.), and *Eisenia fetida* (Savigny) by using hexane (H), chloroform, and ethyl acetate (E) extracts. The chloroform extract exhibited maximum larvicidal activity of 71.55% against *H. armigera* with the least EC_50_ value of 2.95%, followed by ethyl acetate extract which showed larvicidal activity of 40.88% with an EC_50_ value of 5.36%. The lower larvicidal activity was recorded in hexane extract with a higher EC_50_ value of 6.61% concentration. Maximum pupicidal activity of 83.33% was recorded in chloroform extract of *S. surattense* against *H. armigera* and the EC_50_ value was 1.96%. The minimum pupicidal activity of 35.23% was recorded at 5.0% concentration in hexane extract. In the case of EC_50_ value, ethyl acetate extract was the least toxic. At 5.0% concentration, the hexane and ethyl acetate extracts showed statistically similar activities. Based on the bio-efficacy result, the chloroform extract was fractionated into 9 fractions (F) with increasing polarity of the solvent system with hexane, ethyl acetate, and acetone. Among these fractions, F4 (H70: E30) showed the highest effectivity. The F4 showed acute toxicity against *H. armigera* at 1500 and 2000 ppm concentrations. All the concentrations (125, 250, 375, 500 ppm) of F4 showed acute toxicity against *Cx. quinquefasciatus* with an LC_50_ value of 225.70 ppm. None of the concentrations (31.25, 62.5, 125, 250, 500, and 1000 mg/kg dry weight of soil concentration) tested exhibited toxic symptoms or abnormal behavior, nor did they result in mortality of *E. foetida* within the 14-day observation period, and the LC_50_ value was >1000 mg/kg dry weight of soil [162].

The acute toxicity effect of the ethanol extract of *S. surattense* on Swiss albino mice at high concentrations of 100 and 200 mg/kg body weight (oral administration) was reported by Sravanthi et al., 2013 where no changes in the normal behavior of mice, and no signs of toxicity or mortality were observed. In LD_50_ tests, it was found that the animals were safe up to a maximum dose of 2 gm/kg body weight [257]. Gupta et al. (2011) also investigated acute toxicity on Swiss albino mice (6 groups). The ethanolic (50%) extract of *S. surattense* fruit was administered orally at doses of 250, 500, 1000, 1500, and 2000 mg/kg body weight. No mortality was observed at 2000 mg/kg, leading to the selection of 200 mg/kg as the therapeutic middle dose, with 100 mg/kg and 400 mg/kg chosen as the low and high doses, respectively [258].

A study investigated the 95% ethanolic extract of *S. surattense* for acute toxicity and determined the maximum lethal doses for 24 h exposure, where it was determined the doses of 8.64 mg/L and 17.28 mg/L caused 100% mortality to the fish. And this extract also effectively killed mature and young snails at 4.321 mg/L [208]. In another study, *α*-solamargine, one of the active components obtained from the 95% ethanolic extract of *S. surattense* fruit, shows an effective activity of killing (100% at 28 °C) *Oncomelania* snails in an *α*-solamargine solution (0.2 mg/L) [130]. Using in vivo tests on 8 groups of sourian mice, the first 7 groups were infected with *P. berghei* and treated with chloroquine, four different concentrations of *S. surattense* (20, 100, 300, 450 mg/kg), placebo, or no treatment. By day 4, chloroquine completely cleared parasitaemia (0%), while the 450 mg/kg S. surattense group reduced parasitaemia to 4.19%. Placebo and untreated groups showed high parasitaemia (17.2% and 17.8%) with increased levels on day 7. Chloroquine extended survival to 29 days, while the 450 mg/kg *S. surattense* group had a survival of 22 days, compared to 10–14 days in other extract-treated groups. No toxicity was observed in any treatment group [155]. 

The hepatoprotective activity was demonstrated for the 50% ethanolic extracts of *S. surattense* against antitubercular drug (isoniazid (I) 7.5 mg/kg, rifampicin (R) 10 mg/kg and pyrazinamide (P) 35 mg/kg)-induced hepatotoxicity, using various biochemical parameters like serum enzymes: aspartate aminotransferase (AST), alanine aminotransferase (ALT), alkaline phosphatise (ALP), total bilirubin (TBL), albumin (ALB), total protein (TP), lactate dehydroginase (LDH), and serum cholesterol (CHL). Acute liver toxicity resulting from antitubercular drug administration, showed massive fatty changes, focal necrosis with portal inflammation, and loss of cellular boundaries (indicated by the circle) and enhanced the levels of serum enzymes as well as hepatic enzymes. The 50% ethanolic extracts of *S. surattense* (400 mg/kg) inhibited the elevations of these markers by 122.37 ± 5.54, 57.27 ± 5.33, 72.65 ± 6.64, 0.85 ± 0.14, 3.97 ± 0.01, 5.89 ± 0.02, 516.21 ± 3.00, 45.12 ± 2.00, respectively. This was compared with the standard silymarin (100 mg/kg body weight), which reversed the elevation of these markers by 109.89 ± 4.43, 52.84 ± 4.72, 68.34 ± 6.21, 0.83 ± 0.13, 4.68 ± 0.05, 6.22 ± 0.12, 504.23 ± 3.94, and 36.12 ± 1.90, respectively [209]. 

Another study illustrated the protective effects of the ethanolic (50%) extract of *S. surattense* whole plant against Isoniazid and Rifampicin (INH + RIF (50 mg/kg))-induced hepatotoxicity. The extract, at doses of 125 and 250 mg/kg body weight, suppressed the INH + RIF-mediated increase in serum glutamate oxalate transaminase and serum glutamate pyruvate transaminase levels, and restored total bilirubin and alkaline phosphatase to normal values [259]. A similar study found that administering ethanolic (50%) extract of *S. surattense* fruit (100, 200, and 400 mg/kg body weight) daily for 35 days in experimental animals protected against liver toxicity induced by a combination of three antitubercular drugs [isoniazid (INH) 7.5 mg/kg, rifampicin 10 mg/kg, and pyrazinamide (P) 35 mg/kg]. The hepatoprotective activity was assessed using various biochemical parameters, including aspartate aminotransferase, alanine aminotransferase, alkaline phosphatase, total bilirubin, albumin, total protein, lactate dehydrogenase, and serum cholesterol. The results demonstrated that treatment with *S. surattense* significantly (*p* < 0.05–*p* < 0.001) and dose-dependently prevented drug-induced increases in serum levels of hepatic enzymes. Histopathological analysis showed reduced hepatocellular necrosis and inflammatory cell infiltration, indicating the hepatoprotective activity of *S. surattense* [209]. 

The herbicidal effectiveness of *S. surattense* fruit extract was evaluated using a phytotoxic assay on maize seeds with methanolic solutions at 100, 250, 500, and 1000 µg/mL. The extract showed dose-dependent inhibition, with the highest concentration (1000 µg/mL) causing maximum inhibition of root (70.45%) and shoot (63.45%) growth of zea maize. The results demonstrated significant suppression of maize seedling growth compared to the control (methanol) [197].

### 2.8. Other Uses

The study reveals that the species investigated have a significant impact on nutritional value. Mali and Harsh (2014) estimated the protein content of leaf and seed at 11.11% and 12.83%, respectively. Additional findings include carbohydrate content at 75.08% and 71.74%, and crude fiber content at 33.91% and 20.24%. The mineral element composition (mg/100 g) of leaf and seed includes calcium at 1.17 and 1.52, potassium at 0.19 and 0.22, sodium at 0.10 and 0.02, and phosphorus at 0.39 and 0.51, respectively. Phytochemical analysis also revealed high levels of ascorbic acid [44]. Another study estimated total mineral content at 5.093 ± 0.015%, calcium content at 34.44 ± 1.92 mg/100 g, selenium content at 144.80 ± 0.01 mg/100 g, and iron content at 8.87 ± 0.03 mg/100 g [260]. These results indicate that this species has significant nutritional value when used as a vegetable.

Currently, highly toxic and carcinogenic chemicals are used to produce dyes, which harm human health and disrupt ecosystems. The global demand for natural dyes has increased due to their beneficial properties. Tayade et al. (2016) used green techniques to extract dye from *S. surattense* leaf for the finest color. This dye is important for both dyeing and pharmaceutical applications due to its medicinal value [261].

## 3. Materials and Methods

This review was performed following the criteria described in the Preferred Reporting Items for Systematic Reviews and Meta-Analyses (PRISMA) statement 2020 [http://prisma-statement.org/prismastatement/flowdiagram.aspx (accessed on 10 December 2023)]. The PRISMA checklist with detailed information is added as Table S3 in the Supplementary Material.

### 3.1. Search Strategy

This systematic review adheres to PRISMA methodology and analyzes scientific publications between 1753 and 2023 from B-on, Google Scholar, PubMed, Science Direct, and Web of Science. To ensure the quality and reliability of the nonrandomized studies included in this review, each study was assessed using the Newcastle-Ottawa Scale (NOS). This scale evaluates studies based on three broad criteria: the selection of the study groups, the comparability of the groups, and the ascertainment of the exposure or outcome of interest. Each study was scored accordingly to determine its methodological rigor. The explicit mention of NOS ensures that the quality of the nonrandomized studies is systematically evaluated, thereby enhancing the reliability and credibility of the findings presented in the review. *Solanum surattense*, *Solanum xanthocarpum*, ethnomedicinal value, traditional use, phytochemical analysis, and pharmacological activities were used as search keywords.

### 3.2. Data Inclusion and Exclusion Criteria

#### 3.2.1. Inclusion Criteria

- Relevant studies on *S*. *surattense* concerning medicinal importance.
- Full text in English.

#### 3.2.2. Exclusion Criteria

- Duplicate scientific publications;
- Not directly related to the medicinal issues;
- Containing non-relevant or incomplete information.

## 4. Conclusions

This systematic review will play an important role in providing complete knowledge on *S. surattense* as an important natural source of many ethnomedicinal and pharmacological perspectives. It has been used in traditional medicine to cure various ailments since ancient times by traditional practitioners. The literature study indicates the presence of different bioactive secondary metabolites from this species which are very important in both Ayurvedic and modern drug development areas. Additionally, different pharmacological activities have been shown by different plant parts. Systematic investigation ensures that most pharmacological studies were preliminary, carried out in animal models but are not sufficient for the development of a pharmaceutical product. At the present time, alternative drugs as herbal drugs, or herbal drugs with synthetic drugs have become popular for the safety and efficacy of natural products. It could lead to the exploration of new methods for therapeutic and industrial application. So, the present review concludes that the traditional medicinal plant *S. surattense* is a potent source of phytochemicals and pharmacological importance for future pharmaceutical use.

## Figures and Tables

**Table 1:** List of synonyms and local names of *Solanum surattense* [1,9,10,11].

Synonyms	*Solanum arabicum* Dunal; *Solanum armatum* Forssk.; *Solanum ferox* Burm. f.; *Solanum gula* Buch.-Ham.; *Solanum jacquinii* Willd.; *Solanum jacquinii* Miq.; *Solanum macannii* Santapau; *Solanum mairei* H.Lév., *Solanum melongena* Wall.; *Solanum surattense* Burm.f.; *Solanum surattense* var. *awanicum* Yousaf, Mir A.Khan and Shinwari.; *Solanum virginicum* L.; *Solanum xanthocarpum* Schrad.; *Solanum xanthocarpum* var. *geoffrayi* Bonati; *Solanum xanthocarpum* var. *jacquinii* (Willd.) Dunal; *Solanum xanthocarpum* var. *schraderi* Dunal
Local names	kantakari, katabegun (Bangladesh); mao guo qie (China); kateli, oonth kateli, katali, bhatakataiya, chhotikateri, ringani, leipungkhanga, kateringani, kantankattiri, kandan katri, kandanghathiri, kantakariccunta, kantakarivalutana, nelamulaka, vakudu, chinnamulaka, mulaka, pinnamulaka, bhoringan, baiga-kateli, bhurangi, dhaturi etc (India)

**Table 2:** A list polyherbal formulation that includes *S. surattense*.

Formulation Number	Formulation	Ref.
Pf1	*Solanum surattense* whole plant (10%), *Tribulus terrestris* fruit (25%), *Zingiber officinale* root (10%), *Crataeva nurvala* bark (25%) *Tinospora cordifolia* stem (10%) *Asparagus racemosus* root (10%) *Tephrosia purpurea* leaf (10%)	[24]
Pf2	*Solanum surattense* whole plant (25%), *Piper longum* fruit (10%), *Adhatoda vasica* leaf (25%), *Zingiber officinale* root (10%), *Curcuma zedoaria* root (10%), *Ocimum sanctum* leaf (10%), *Phyllanthus emblica* fruit (10%)	[24]
Pf3	*Solanum surattense* whole plant (15%), *Piper longum* fruit (10%), *Withania somnifera* root (10%), *Terminalia chebula* fruit (10%), *T. bellerica* fruit (10%), *Curcuma zedoaria* root (15%), *Phyllanthus emblica* fruit (15%), *Ricinus communis* root (15%)	[24]
Pf4	*Solanum surattense* whole plant (10%), *Phyllanthus emblica* fruit (25%), *Adhatoda vasica* leaf (20%), *Ocimum sanctum* leaf (10%), *Piper longum* fruit (10%), *Zingiber officinale* root (10%), *Glycyrrhiza glabra* root (15%)	[24]
Pf5	*Solanum surattense* whole plant (20%), *Glycyrrhiza glabra* root (30%), *Terminalia chebula* fruit (10%), *T. bellerica* fruit (10%), *Piper longum* fruit (10%), *Sida cordifolia* root (10%), *Phyllanthus emblica* fruit (10%)	[24]
Pf6	*Solanum surattense* root (50 g), *Oroxylum indicum* bark (100 g) and flowers (3), *Ocimum tenuiflorum* leaf (10–15), *Clerodendrum villosum* root (50 g) and flowers (10–15), *Curcuma aromatica* rhizome (50 g), *Musa paradisiaca* root (25 g) and *Breonia chinensis* leaf (3)	[91]
Pf7	*Solanum surattense* root (½ kg) and *Saccharum bengalense* Retz. root (½ kg)	[71]
Pf8	*Solanum surattense* root (5 g) and a whole mature plant of *Andrographis paniculata* along with black pepper (10 g) and Ajwain (*Trachyspermum ammi* L.) (10 g)	[98]
Pf9 (Dasamula)	A mixture of 10 roots of different species (*Solanum surattense*, *Aegle marmelos*, *Premna obtusifolia/Clerodendrum phlomidis*, *Gmelina arborea*, *Oroxylum indicum*, *Stereospermum suaveolens*, *Desmodium gangeticum*, *Uraria picta*, *Solanum indica*, and *Tribulus terrestris*)	[102]

Pf—Polyherbal formulations.

**Table 3:** Quantitative phytochemical estimation of different extracts of *S. surattense*.

Species	Phytochemical Content	Plant Part	Extract	Results	Ref.
*S. surattense*	Total phenolic contents	L	ethanol	46.7 GAE/mg	[146]
			methanol	25.9 GAE/mg	[147]
			methanol	14.7 Pmol GA/ug	[148]
			methanol	28.3 ± 2.0 GAE/mg	[113]
			acetone	26.2 ± 1.5 GAE/mg	[113]
			ethyl acetate	23.2 ± 1.2 GAE/mg	[113]
			chloroform	24.2 ± 1.0 GAE/mg	[113]
			hexane	21.2 ± 1.7 GAE/mg	[113]
		F	methanol	12.3 ± 1.73 Pmol GA/ug	[148]
			methanol	7.6 ± 0.3 (for raw) and 6.1 ± 0.3 (for boiled) g/100 g	[149]
			methanol	4.975 GAE/mg	[147]
		St	methanol	5.87 GAE/mg	[147]
		Stb	methanol	21.1 ± 2.88 Pmol GA/ug	[148]
		R	acetone	28.9 g/100 g	[150]
		Rb	methanol	23.2 ±1.3 Pmol GA/ug	[148]
	Total flavonoid contents	L	methanol	17.7 (QE)/mg	[147]
			methanol	25.2 ± 1.2 Rutin/µg	[113]
			acetone	17.1 ± 0.8 Rutin/µg	[113]
			ethyl acetate	22.1 ± 0.5 Rutin/µg	[113]
			chloroform	16.5 ± 1.3 Rutin/µg	[113]
			hexane	23.2 ± 1.0 Rutin/µg	[113]
			methanol methanol	2.48 ± 0.6 Rutin/µg	[148]
		F		5.21 QE/mg	[147]
			hexane	71.8 ± 0.08 μg QE/mg	[151]
			benzene	69.7 ± 0.12 μg QE/mg	[151]
			chloroform	59.5 ± 0.13 μg QE/mg	[151]
			ethyl acetate	162.4 ± 0.15 μg QE/mg	[151]
			acetone	148 ± 0.18 μg QE/mg	[151]
			ethyl alcohol	71.4 ± 0.14 μg QE/mg	[151]
			aqueous	10.2 ± 0.12 μg QE/mg	[151]
			methanol	8.33 ± 1.7 Rutin/µg	[148]
		St	methanol	3.129 QE/mg	[147]
		Stb Rb	methanol	15.3 ± 2.3 Rutin/µg 17.8 ± 1.7 Rutin/µg	[148]
			methanol		[148]
	Total tannin contents	F	methanol acetone	7.0 ± 0.4 (for raw) and 5.6 ± 0.4 (for boiled) g/100 g 18.7 g/100 g extract	[149] [150]
		R			
	Total terpenoid contents	L	methanol	6.3 ± 1.2 GAE/mg	[113]
			acetone	6.1 ± 1.0 GAE/mg	[113]
			ethyl acetate	5.7 ± 0.3 GAE/mg	[113]
			chloroform	4.5 ± 1.0 GAE/mg	[113]
			hexane	5.2 ± 1.4 GAE/mg	[113]

L—leaf, F—fruit, St—stem, Stb—stem bark, R—root, Rb—root bark.

**Table 4:** In vitro and in vivo pharmacological and toxicological studies based on *S. surattense*.

Plant Part	Solvent/Compound	Biological Activity	Model Organisms	Study Design	Assay/Route of Administration	Tested Concentration	Results	Ref.
Wp	methanol, ethanol, chloroform	antimicrobial	bacteria (*Staphylococcus cohnii*)	in vitro	disk diffusion	25, 50, 75, 100 µL	MIC values of 0.06 mg/mL in methanol extract, 0.51 mg/mL in ethanol, and 0.60 mg/mL in chloroform extract	[153]
	aqueous	antimicrobial	bacteria (*Bacillus subtilis*, *S. aureus*, *E. coli*, *P. aeruginosa*)	in vitro	disk diffusion	125, 250, 500 and 1000 mg/mL	the highest antimicrobial activity showed in highest concentration after 24 h exposures	[154]
		antioxidant		in vitro	DPPH radical scavenging activity	0.25, 0.50, 1.0 and 2.0 mg per 10 mL	percentage of inhibition of free radicals is dose dependent	
	ethanol	anti-malarial	mice	in vivo	parasite lactate dehydrogenase (pLDH)	20, 100, 300, and 450 mg/kg	concentration of 450 mg/kg showed a significant impact in reducing parasitaemia in infected mice (*p* < 0.05)	[155]
	methanol 70%	antidepressant	mice	in vivo	oral administration	100–200 mg/kg	reduce immobility time, influence the antidepressant effect	[156]
	methanol	antimicrobial	bacteria (*E. coli*)	in vitro	disc diffusion	1 mg/mL	inhibition zone 14.8 ± 0.5 mm	[157]
	ethanol	antipyretic	albino rats	in vivo	oral administration	250–600 mg/kg	significant antipyretic effects were observed, which were comparable to standard, aspirin	[62]
	acetone, methanol	phytotoxic	*Lemna minor*	in vitro	growth inhibitor (paraquate)	10–1000 µg/mL	significant activity showed at 10 µg/mL to 100 µg/mL	[158]
Wp		cytotoxic	*Artemia salina*	in vitro	brine shrimp lethality	10, 100 and 1000 µg/mL	acetone extracts showed a very low cytotoxic effect	
	ethanol, hydroethanol	antimicrobial	bacteria (*E. coil* MTCC 2960, *P. auruginosssa* MTTC 4676, *S. aureus* MTTC 3160, *Klebsiella oxytoca* MTTC 3030, *B. subtilis* MTCC 1790), fungi (*Candida albicans* MTCC 183)	in vitro	agar well diffusion	50 μg/mL	ethanol extract was found to be having more potent anti-microbial activity than hydroethanol extract	[159]
		antioxidant		in vitro	FRAP and DPPH assays		mild activity	
		anthelmintic	*Eisenia fetida*	in vitro	adult motility assay (AMA)	100 mg/mL	ethanol extract was having more significant than hydroethanolic extract	
	methanol	anti-constipation and anti-diarrheal	rabbit (jejunum)	in vivo		3–5 mg/mL	EC_50_ value 3.17	[160]
	aqueous	estrogenic	albino rats	in vivo	oral administered	200 mg/kg	significantly improved all the parameters of sexual behavior (*p* < 0.01), caused vaginal cornification, and increased serum estradiol and uterine weight	[161]
	hexane: ethyl acetate (70:30)	pupicidal	*Helicoverpa armigera*	in vitro	dose mortality test	125, 250, 375 and 500 mg/L	pupicidal activity against *H. armigera* with EC_50_ value of 345.34 mg/L at 1000 mg/L	[162]
Wp		larvicidal	*Culex quinquefasciatus*	in vitro	dose mortality test	125, 250, 375 and 500 mg/L	maximum larvicidal activity against *C. quinquefasciatus* with LC_50_ value of 225.70 mg/L at 500 mg/L	
	methanol	cytotoxic	NIH-3T3 fibroblast cancer cell line	in vitro	MTT assay	30 μg/mL	showed strong cytotoxicity against 3T3 cell line	[163]
	methanol and aqueous	anticonvulsant	albino mice (male)	in vivo	maximum electric shock (MES), pentylenetetrazole (PTZ) induced methods/oral administration	200 mg/kg b.w.	showed significant activity in MES induced seizures by reducing tonic hind limb extension (7.16 ± 0.47 s and 0.17 ± 0.47 s); and delayed the onset of clonus (92.33 ± 1.66 s, 86.33 ±0.49 s) induced by PTZ	[164]
	ethanol	antimicrobial	fungi (*Enterobacter aerogenes*, *C. albicians*, *A. niger*)	in vitro	disc Diffusion Method	1, 5, and 10 mg	maximum activity was shown against *E. aerogenes* (10 mm); *C. albicians* (10 mm) and *A. niger* (7.6 mm)	[165]
Wp (except root)	methanol	antimicrobial	fungi (*Trichophyton rubrum*, *C. albicans*, and *Epidermophyton floccosum*)	in vitro	plate hole diffusion	Con. 5–25%	mycelial inhibition up to 18 ± 1.3 to 0.3 03± 0.4 mm on *T. rubrum*, 16 ± 0.8 to 00 ± 0.0 mm on *C. albicans*, and 20 ± 1.1 to 00 ± 0.0 mm on *E. floccosum*	[166]
		antioxidant		in vitro	DPPH		significant antioxidant activity with IC_50_ = 10.15 g/mL	
		nephroprotective	human embryonic kidney cell lines (HEK293)	in vitro	MTT	50–500 μg/mL	protect up to 95.31% of human embryonic kidney-293 cells from cisplatin nephrotoxicity	
Ap	alcohol	anti-inflammatory	albino rats	in vivo	topical and oral administration	200 mg/kg	the highest efficacy in healing was observed at 10% gel (topical) and 200 mg/kg (orally) in diabetic rats, where the maximum healing power was observed when treated both orally and topically	[167]
	ethyl acetate	anti-malarial	3D7 and INDO strains	in vitro		100 μg/mL	resistant INDO, IC_50_—7 μg/mL and sensitive 3D7 IC_50_—17 μg/mL and TC_50_—75 μg/mL	[168]
		cytotoxic	hela cell line	in vitro	MTT assay	not mentioned		
	ethanol	antimicrobial	bacteria (*E. coli*, *S. aureus*, *Salmonella typhi*, *P. aeruginosa* and *Serratia marcescens*)	in vitro		30 mg (concentration)	extract showed the activity against only *E. coli* (10.10 ± 0.91) mm	[169]
	ethanol	anti-malarial	*P. falciparum* K1 (chloroquine-resistant strain) and CY27 (chloroquine-sensitive strain) and *P. berghei* (ANKA strain)	in vitro	parasite lactate dehydrogenase (pLDH) assay	50 μg/mL	IC_50_ ≤ 50 μg/mL for K1; 40.88 μg/mL for CY27	[31]
Ap	alcohol	antidepressant	albino mice	in vitro and in vivo	TST and FST/oral administration	50 and 100 mg/kg p.o.	decreased the immobility periods significantly in a dose-dependent manner in both TST and FST, showing significant antidepressant-like activity	[170]
L	aqueous, powder	cytotoxic	MCF cell line	in vitro	MTT assay	200, 400, 600, 800, and 1000 µg/dL	50% reduction in the viability of cancer cells at con. of 62.5 and 31.2 µg/mL for aqueous and powder extract	[171]
	aqueous, powder	anti-obesity	porcine pancreatic lipase enzyme	in vitro	pancreatic lipase inhibition assay	200, 400, 600, 800, and 1000 µg/dL	pancreatic lipase inhibitory activity of the dry and fresh leaf extract showed in a dose-dependent manner	
	100 mL of sterile distilled water; 1 mM auric chloride solution	cytotoxic	C666-1 cell line (nasopharyngeal cancer (NPC) cell line)	in vitro	MTT and TUNEL	15 µg/mL	significant decrease in viability of C666-1 cells upon treatment with 15 µg/mL Sx-AuNPs by autophagy and mitochondrial-dependent apoptotic pathway	[172]
	methanol	antidiabetic	albino rat	in vivo		100–200 mg/kg bw	showed efficient anti-hyperglycemic activity at a con. of 200 mg/kg b.w.	[173]
		antioxidant		in vitro			enhanced the level of antioxidant enzymes catalase (CAT), superoxide dismutase (SOD), and glutathione (GSH) peroxidase in alloxan-induced animal models	
L	petroleum, ethanol, chloroform	antiulcer		in vivo		100 mg/kg	significant results showed as antiulcer potential	[174]
	methanol	analgesic, anti-inflammatory, and anxiolytic	albino mice	in vivo	oral administration	100–200 mg/kg	effective against analgesic as well as anti-inflammatory activity but did not ensure anxiolytic and tranquilizing activity except at high doses	[175]
	ethanol, chloroform, methanol, acetone	molluscicidal	*Lymnaea acuminata*	in vitro	mortality test	157.33 mg/L and 150.26 mg/L	ethanol extract of dried leaf powder was found more toxic; LC_50_ was 157.33 mg/L and at 96h 150.26 mg/L	[176]
	ethanol	antidiabetic	albino rats	in vivo	oral administration	100, 200 and 300 mg/kg	significantly decrease the glucose level in blood and an increase in plasma insulin level at 100 mg/kg	[177]
	ethanol	antidiabetic and anti-hyperlipidemic	albino rats	in vivo	oral administration	100 mg/kg b.w.	significantly increase in plasma insulin; control the function of T), TG, PL and FFA in the plasma; increase HDL-C as well as maintained the level of levels of linolenic and arachidonic acids	[178]
	ethanol	antimicrobial	bacteria (*S. aureus*, *Streptococcus* sp.; *B. subtilis*, *E. coli*, *P. aeruginosa*, *S. typhi*, *Shigella dysenteriae* and *V. cholerae*)	in vitro	agar-well diffusion method	50–500 µg/mL	maximum zone of inhibition was observed in 500 µg concentration of leaf extract of all bacteria screened except *S. dysenteriae*	[179]
L	ethanol	antioxidant		in vitro	hydroxyl radical scavenging	50–250 μg/mL	(IC_50_ value 154.03 μg/mL)	[146]
				in vitro	scavenging of hydrogen peroxide	50–250 μg/mL	(IC_50_ value 147.23 μg/mL)	
				in vitro	superoxide anion scavenging activity	50–250 μg/mL	(IC_50_ value 145.22 μg/mL)	
				in vitro	DPPH	20–100 μg/mL	(IC_50_ value 55.62 μg/mL)	
				in vitro	ABTS	20–160 μg/mL	(IC_50_ value 89.28 μg/mL)	
	aqueous	antitussive	guinea pig	in vivo	oral administration	25 mg/kg	provides a molecular entity, that induces antitussive activity	[180]
	methanol	antioxidant		in vitro	DPPH	different conc.	highest radical scavenging effect was observed with IC_50_ = 22.936 ± 2.685 µg/mL	[147]
	silver nanoparticle solution	cytotoxic	MCF-7 cancer Cell line	in vitro	MTT	50 µg/mL	toxic activity showed against MCF-7	[181]
	ethanol	hepatoprotective	albino rats	in vivo	intragastric intubation (oral)	150 mg/kg b.w.	to prevent tumor incidence and restored the elevated activities of liver marker enzymes and antioxidant status to near normal with decreased lipid peroxide levels	[182]
	hexane, acetone, ethyl acetate, chloroform, and methanol	antimicrobial	bacteria (*E. coli*, *S. aureus*, *S. typhi*, *P. aeruginosa*)	in vitro	agar well diffusion	100 μg/mL (dissolved in 10% DMSO)	methanol extract showed significant inhibitory effect against *P. aeruginosa* (12 ± 0.5 mm), *S. typhi* (10 ± 0.6 mm), *S. aureus* (9 ± 1.0 mm), and *E. coli* (7 ± 1.3 mm)	[113]
L		antioxidant		in vitro	DPPH	20, 40, 60, 80, and 100 μL/mL	antioxidant activity was observed in chloroform and methanol extract on the DPPH radical scavenging activity with the lowest IC_50_ value of 197.245 μg/mL (chloro-form) and 201.04 μg/mL (methanol)	
	methanol	antioxidant		in vitro	DPPH	5–25 mg/mL	significant free radical scavenging activity showed with IC_50_ value 11.72 μg	[183]
				in vitro	ABTS		highest ABTS radical scavenging activity (IC_50_, 17.99 μg)	
		hepatoprotective	albino rats	in vivo	oral administration	100 and 200 mg/kg b.w.	significantly enhanced levels of SOD (1.78 ± 0.13), CAT (34.63 ± 1.98), GST (231.64 ± 14.28), and GSH (8.23 ± 0.48) in liver homogenates	
	ethanol	hepatoprotective	HepG2 cells	in vitro	MTT	50–200 μg/mL	hepatoprotective and anti-apoptotic potential against chemical-induced liver damage	[184]
		anti-apoptotic			Caspase-3/7	50–200 μg/mL		
	ethanol	diuretic	albino rats	in vivo	oral administration	500–1000 mg/kg	increase total urine volume and levels of sodium, potassium, and chloride	[185]
		anti-inflammatory	albino rats	in vivo	oral administration	500–1000 mg/kg	reduced paw edema	
L	aqueous, hexanic	antimicrobial	fungi (*A. niger* and *C. albicans*)	in vitro	agar well-diffusion	100–500 µg/mL	maximum inhibition zone 2.5 and 6 mm at 500 µg/mL was found in hexane extract	[186]
	methanol and acetone	antimicrobial	bacteria (*E. coli*, *Yersinia pestis*, *P. aeruginosa*, *S. aureus*)	in vitro	agar well diffusion method	Con.30, 50, 70 and 100%	methanolic and acetone extract were most effective against *S. aureus* (18 and 16 mm) and minimum inhibition in *P. aeruginosa* (13 and 12 mm) at 100%	[187]
	aqueous, ethanol, acetone, methanol	antimicrobial	bacteria (*S. aureus*, *S. pyrogens*, *S.mutans*, *B. Sphaericus*, *S. parathypi*, *E. coli*, *P. aeru-ginosa*, *Proteus vulgaris*, *K. pneumoniae*, *S. marcescens*)	in vitro	agar well diffusion method	75, 50, 25, and 10 mg/mL	ethyl acetate extract exhibited highest degree of activity against *S. pyrogens* (26 mm, 26 mm), aqueous extracts of leaf (field grown, tissue cultured) showed the highest inhibition against *E. coli* (20 mm, 18 mm)	[188]
		anti-inflammatory	albino rat	in vivo	excision and incision wound/topically applied	10% *w*/*v*	reduced the epithelization period and the scar area, and increased tensile strength of control and ethanol extract, respectively, and results showed in significant	[189]
	ethanol (95%)	anti-asthmatic	guinea pigs	in vivo	histamine and acetylcholine-induced bronchospasm	50, 100, 200, 300 mg/kg b.w.	200 and 300 mg/kg have shown significant bronchoprotection (80 and 70%) against histamine, but not on acetylcholine	[190]
L	ethanol (95%)	anti-inflammatory	sprague–dawley rats	in vivo	histamine, carrageenan, dextran, formaldehyde—induced hind paw edema; cotton pellet granuloma	50, 100, 200 and 400 mg/kg	significantly reduced the paw edema at the dose of 200 and 300 mg/kg b.w in all assays (inhibition (%) range 45.25 to 61.11)	
L (shoot)	methanol	antioxidant		in vitro	DPPH (2,2-diphenyl-1-picrylhydrazyl) assay	10 mg/L	The highest DPPH antioxidant values were observed at 10 mg/L of TDZ (94.6 ± 2.29% RSA), 2.5 mg/L of BAP (92.6 ± 3.10% RSA), and 5 mg/L of TDZ (92 ± 3.49% RSA), whereas the lowest DPPH value was	[191]
					FRAP (ferric antioxidant power) assay	2.5 mg/L	recorded at 10 mg/L of NAA (71.4% RSA); maximum FRAP and ABTS antioxidant activities were obtained with 5 mg/L of TDZ (654 ± 5.39 µM TEAC and 402.5 ± 5.16 µM TEAC, respectively)	
					ABTS (2,2′-azino-bis(3-ethylbenzothiazoline-6-sulfonic acid) assay	5 mg/L		
		antiaging			inhibition of AGE formation	5 mg/L (AGEs-Vesper lysine); 2.5 mg/L (AGEs-Pentosidine)	The highest inhibition against vesper lysine-like AGEs (51.55 ± 2.67%) and the formation and inhibition of tyrosinase (32.87 ± 2.04%) and collagenase (49.52 ± 2.69%) enzymes, while the extract obtained from the	
					Tyrosinase—inhibition	5 mg/L		
L (shoot)					Elastase—inhibition	1.0 mg/L	callus treated with 2.5 mg/L of TDZ showed the maximum inhibition (59.75 ± 3.15) against pentosidine-like AGE formation	
		anti-inflammatory	COX2 (human) and COX1 (ovine) enzymes	in vitro	secretory phospholipase (sPLA2)—Inhibition	0.1 mg/L	greatest anti-inflammatory action was found for: the extract obtained from the *S. virginianum* callus culture treated with 0.1 mg/L of TDZ (11.3 ± 1.02%) against sPLA2; 5 mg/L of TDZ (38.5 ± 2.29%) against 15-LOX; 10 mg/L of TDZ (38.06 ± 2.49%) against COX-1; and 2.5 mg/L of TDZ (15.5 ± 0.71%) against COX-2	
					15-lipoxygenase (15-LOX)	5 mg/L		
					cyclooxygenases 1 and 2 (COX1 and COX2)	10 mg/L (1); 2.5 mg/L (2)		
Fl	ethanol (95%)	anti-asthmatic	male albino mice	in vivo	milk- induced eosinophilia method	100 mg/kg	significantly (*p* < 0.05) reduced milk induced eosinophilia (18.16 ± 0.912)	[192]
				in vivo	mast cell degranulation	25, 50 and 100 mg/kg	mast cells were protected at a dose of 50 and 100 mg/kg by 74.39% and 78.26%, respectively	
				in vivo	capillary permeability	25, 50 and 100 mg/kg	decrease in intestinal capillary permeability of 62%	
	aqueous	antagonistic	goat tracheal chain	in vivo	sensitivity	2, 4 and 10 mg/mL	at a dose of 10 mg/mL (44.71 ± 0.947) exhibited significant antagonistic effect	
F	ethanol	hepatoprotective	albino rats	in vivo	oral administration	200 and 400 mg/kg	combination of *S. surattense* and *Juniperus communis* showed significant hepatoprotective potential against AZM and PCM induced liver toxicity	[193]
	methanol	antiurolithiatic	albino rats (male)	in vivo	oral administration	100, 200, and 400 mg/kg	reduced and prevented the growth of urinary stones and maintaining balance between stone promoters and inhibitors constituents	[194]
	petroleum ether, chloroform, dichloromethane, ethyl acetate, acetone, methanol and aqueous	antimicrobial	bacteria (*Micrococcus varians*, *M. luteus*, S. *aureus*, *Pasteurella maltocida*, *S. typhi*, *E. coli*); fungi (*A. niger*, *A. flavus*, *A. fumigatus*)	in vitro	hole-plate diffusion method	5, 10 and 15 mg/mL	significant zones of inhibition were showed against all organisoms in case of MeOH extract	[195]
	methanol	antimicrobial	bacteria (*A. niger*, *Trichoderma viride*)	in vitro		NT	exhibited inhibitory effects on the radial growth of *A. niger* and *T. viride*	[196]
	ethanol	anti-rheumatic	chondrocyte cells	in vitro	MTT assay	25, 50, 100, 250, 500 μg/mL	enhanced the cell proliferation in a dose-dependent manner and has no cytotoxic effect on primary chondrocytes	[115]
		anti-rheumatic	sprague dawley rats	in vivo	oral administration	250 and 500 mg/kg body wt.	restored the synthesis of collagen and proteoglycan, vital factors for cartilage restoration, and reduced the arthritic score, and protect the cartilage destruction	
F	ethanol–aqueous (1:1)	antiurolithiatic	albino rats	in vivo		20 and 40 mg/kg	simultaneous administration of SXS, prevent renal tissue and cellular injury, decreased antioxidant enzyme catalase activities of the kidneys and raised level of glycosaminoglycan, a stone inhibitor	[194]
				in vitro	nucleation and aggregation	10–100 μg/m		
	methanol extract (80%)	cytotoxic	*A. salina*	in vitro	brine shrimp lethality bioassay	100, 250, 500, 1000 µg/mL	significant cytotoxicity showed at concentration of 500 µg/mL	[197]
		antioxidant		in vitro	DPPH assay	50–500 µg/mL	significant antioxidant showed	
	petroleum ether, ethanol	anti-inflammatory	human red blood cell (HRBC)	in vitro	HRBC membrane stabilizing activity assay	1–6 mg/mL	ethanol extract at concentration 6 mg/mL showed 50.1% protection of HRBC in hypotonic solution	[198]
	ethanol	antihelmenthic	*Pheritima posthuma*		in vitro/adult motility assay (AMA)	10, 25, 50 mg/mL	paralyzed and cause of death at a concentration of 10 and 50 mg/mL	[199]
	aqueous, ethanol	antihelmenthic	*Pheritima posthuma*		in vitro/adult motility assay (AMA)	10, 15, 20 mg/mL, 25 mg/mL, 30 mg/mL and 35 mg/mL	water extract showed better anthelmintic activity in comparison to the Ethanol extract	[200]
	ethanol (50%)	nephroprotective	albino rats		in vivo/intraperitoneal administration	200 and 400 mg/kg/d	acts as a potent scavenger of free radicals to prevent the toxic effects of gentamicin both in the biochemical and histopathological parameters	[201]
	aqueous	hypoglycemic	albino rats		in vivo/oral administration	100 and 200 mg/kg	exhibited a potent blood glucose-lowering property	[202]
F	ethanol (50%)	hepatoprotective	sprague-dawley rat; albino mice		in vivo/oral administration	400 mg/kg	extract significantly (up to *p* < 0.001) reduced the lipid peroxidation in the liver tissue and restored activities of defence antioxidant enzymes GSH, SOD and catalase towards normal levels	[203]
	hexane, benzene, chloroform, ethyl acetate, acetone, ethyl alcohol and aqueous	antioxidant			DPPH radical scavenging assay	250, 500, 1000 μg/mL	antioxidant activities of the extracts varied significantly with different concentrations	[151]
		cytotoxic	lungs (HOP-62) and leukemia (THP-1) cell lines	in vitro	sulforhodamine-B assay	100 μL test extract in DMSO (100 μg/well)	cytotoxic potential showed against HOP-62 (lung) and THP-1 (leukemia) human cancer cell lines in presence of 100 μg of extract per weel	
		antimicrobial	virus (HIV)		RT assay kit (Roche)	0.6 and 6.0 μg/mL.	inhibitory activity was observed in non-polar extracts against HIV reverse transcriptase enzyme	
	aqueous, ethanol	cytotoxic	HepG2 cell line	in vitro	antiproliferative assay	200–400 mg/mL	ethanol extract revealed higher cytotoxicity (49.25 ± 0.38–73.2 ± 0.3%) than the aqueous extract (32.23 ± 0.34–54.82 ± 0.26%) with significant morphological changes	[118]
		antioxidant		in vitro	DPPH assay; ABTS^+^ assay; Ferric (Fe^3+^) reduction assay	20–120 mg/mL; 2–12 mg/mL; 20–120 mg/mL	all extracts showed significant scavenging of DPPH, ABTS^+^ radicals and also in ferric reducing power	
F		antidiabetic		in vitro	Starch-iodine assay	20–120 mg/mL	water extract (54.12 ± 0.44–86.80 ± 0.27%) higher rate of a-amylase inhibition than ethanol extract (23.07 ± 0.47–81.61 ± 0.43%)	
	aqueous and ethanol	anti-inflammatory		in vitro		20–100 mg/mL	hemolysis inhibition (46.19 ± 0.14–66.21 ± 0.17%) higher than the ethanol extract (12.67 ± 0.19–38.03 ± 0.41%) while diclofenac showed (48.26 ± 0.11–70.39 ± 0.28%)	
	ethanol	antimicrobial	bacteria (*S. aureus*)	in vitro		500 mg/mL	MIZ = 22.3–0.6 mm	
	aqueous	anti-inflammatory	albino rat	in vivo	carrageenan-induced paw edema model/oral administration	500 mg/kg	showed the maximum percentage inhibition of 75%, which was comparable with the positive standard diclofenac synergistic effect	[204]
		anthelmintic	*Pheretima posthuma*		used directly (surface film method)	10 mg/mL	*Solanum surattense* exhibited greater anthelmintic activity that aqueous extract	[205]
	methanol (70%)	diuretic	albino rats	in vivo	administered intraperitoneally	100 mg/kg	significantly increased the urinary electrolyte excretion, especially calcium (*p* < 0.05); urine volume = 2.72 ± 0.09 mL	[206]
	ethanol	molluscicidal	*Biomphalaria glabrata* Say and *Indoplanorbis exustus*		dose mortality	125, 150, 175, 200, 225 and 250 mg/L	LC_50_ against *B. glabrata* and *I. exustus* were reported at 163.85 and 198.00 mg/L while LC_90_ were 219.33 and 236.80 mg/L, respectively	[207]
F		larvicidal	*Aedes aegypti and C. quinquefasciatus*			125, 150, 175, 200, 225 and 250 mg/L	LC_50_ against *A. aegypti* and *C. quinquefasciatus* were 788.10 and 573.20 mg/l, while LC_90_ were 1288.91 and 1066.93 mg/L, respectively	
	methanol	larvicidal	*A. aegypti*		dose mortality	100, 150, 200, 250, and 300 mg/L	LC_50_ and LC_90_ against the first to fourth instar larvae and pupae were 170.91, 195.07, 221.45, 253.18, and 279.52 mg/l and 320.62, 366.48, 410.20, 435.16, and 462.10 mg/l, respectively	[208]
	ethanol	molluscicidal	*Oncomelania hupensis*, *B. glabrata*, *Lymnaea stagnalis* L.		dose mortality	0.0675–8.640 mg/L	significant toxicity showed with the LC_50_ value of 0.332, 0.858 and 0.747 mg/L	[209]
	ethanol (50%)	hepatoprotective	albino mice	in vivo	oral administration	100, 200 and 400 mg/kg bw	showed that attenuated the hepatocellular necrosis and led to reduction of inflammatory cells infiltration at 400 mg/kg	[210]
	ethanol, aqueous	hepatoprotective	albino rats (male)	in vivo	oral administration	2 mL/kg (for 7 days)	alcoholic and aqueous extract showed significant (*p* < 0.001) reduction in serum marker enzymes and antioxidant levels to near normal against CCl4-induced rats and protected liver from CCl4 damage	[211]
F	aqueous	hypoglycaemic	albino rats	in vivo	oral administration	100 and 200 mg/kg	decrease the glucose level in normoglycemic, alloxan induced diabetic and glucose loaded hyperglycaemic rats at 100 and 200 mg/kg, with the value of 78.98 ± 2.18 and 68.21 ± 3.0 mg/dL; 128.47 ± 6.27 and 109.34 ± 5.91 mg/dL and 93.0 ± 4.24 and 83.5 ± 2.12 mg/dL, respectively, after 10 h exposures	[212]
	aqueous, hexane, ethyl acetate, chloroform, and ethanol.	antimicrobial	bacteria (*Micrococcus varians*, *M. luteus*, and *S. aureus*, *S. typhi*, *Pasteurella multocida*, *E. coli*, *K. pneumoniae*, *V. cholerae*); fungi (*A. niger*, *A. flavus*, *A. fumigatus*)	In vitro	dental plaque biofilm	25, 50, 75, and 100 mg/mL	maximum inhibition of microbial growth, MIC was evaluated at 0.625 g/mL against all microbial agents	[213]
	methanol	antiurolithiatic	albino rats	in vivo	electrolyte flame photometery/oral administration	40 to 300 mg/kg b. W.	showed significant antiurolithiatic activity at 80 mg/kg b.w.	[214]
	aqueous/ethanol	anthelmintic	*P. posthuma*	in vitro		25, 50, and 100 mg/mL	produced paralysis as well as death of worms in a less time at higher concentration of 100 mg/mL	[215]
F	methanol	anti-inflammatory	sprague-dowlay rat	in vivo	excision and incision wound/topically applied	10% *w*/*w*	effectively increased (30%) the contraction of open wound, tensile strength (37.5%), and significantly (*p* < 0.01) enhanced the wound healing process	[216]
	aqueous (normal and boiled)	antioxidant		in vitro	DPPH assay	1.9 g extract/g DPPH	exhibited good scavenging, reducing potentiality with the value of 2.1 ± 0.2 and 2.5 ± 0.0 (boiled) g extract/g	[147]
					ABTS+ assay	236.1 µmol/g	exhibited good scavenging reducing potentiality with the value of 7.0 ± 0.0 and 28.5 ± 0.0 (boiled) µg extract/mmol fe(ii)	
S	aqueous	oxidative potential	albino rats	in vivo	oral administration	10 mg/kg b.w.	depleted the oxidative stress of cauda epididymal spermatozoa	[217]
	aqueous, ethanol and methanol	antimicrobial	fungi (*C. albicans*, *C. tropicalis*, *C. krusei*, *C. kefyr*, *A. niger*, *A. fumigates*, *A. flavus*, *Rhizopus oryzae*)	in vitro	agar-well diffusion method		ethanol seed extracts showed high antimicrobial activity against *C. albicans*, *C. tropicalis*, *A. niger*, *A. fumigates* and *A. flavus*; in methanol extracts showed activity against *A. fumigatus* and *R. oryzae*; and aqueous extracts showed *C. albicans* but did not show activity against *C. tropicalis*, *C. krusei*, *C. kefyr*, *A. niger*, *A. fumigatus*, *A. flavus*, *R. oryzae*	[218]
S	ethanol	analgesic	human (*Homo sapiens*)	in vivo	mouth rinse	5 g/50 mL	results showed 68% reduction in pulpal pain	[219]
St	ethanol	anti-psoriatic	albino mice		oral and topical	10% (topical) and 200 and 400 mg/kg (oral)	significant inhibition in the expression of TNF-α, IL-1β, IL-6 and IL-17 in treated animal tissues; also showed significant restoration of the altered biochemical parameters along with reduced hyperkeratinisation; the effect was found to be more prominent topically than orally	[220]
Stb	methanol	antidiabetic and antioxidant	albino rats	in vivo	oral administration	10, 15 and 20 mg⁄kg (β-sitosterol)	resulted in decreased inglycated hemoglobin, serum glucose, and nitric oxide, with concomitant increases in serum insulin level; treatment with BS doses also increased pancreatic antioxidant levels	[221]
B	methanol	leishmanicidal	*Leishmania tropica*			45 mg/mL (after 96 h)	showed significant leishmanicidal, antioxidant and anti-microbial potential activity	[222]
		antioxidant	DPPH	in vitro		effective scavenging concentrations (50 to 500) μg/mL		
		antimicrobial	bacteria (*S. aureus*, *E. coli* and *K. pneumoniae*)	in vitro		1 mg/m		
R	ethanol	antidiabetic	albino rats	in vivo	intraperitoneal administered	200–400 mg/kg	EESS root elicited significant (*p* < 0.01) reductions of blood glucose, lipid parameters and serum enzymes and significant (*p* < 0.01) reductions of blood glucose	[223]
	methanol	antimicrobial	fungi (*C. albicans*, *C. glabrata*, *C. krusei* and *C. tropicalis*)	in vitro	agar well diffusion method	20–80 μL/well	methanol root extract showed significant activity	[224]
	ethyl acetate, chloroform	antimetastatic, cytotoxic	carcinoma A549 cell line	in vitro	wound healing scratch; MTT assay; $NO_{2}^{-}$ production in A549 cells; superoxide anion determination (NTB)	20 μg/mL (MTT assay)	wound healing efficiently inhibited migration of A549 cells as well as $NO_{2}^{-}$ and decreased levels of $O_{2}^{-}$	[225]
	ethyl acetate, citrulline and chloramphenicol	antimicrobial	bacteria (*Ralstonia solanacearum*) and fungi (*Fusarium oxysporum*)	in silico and in vitro	dual-plate technique	1–10 mg/mL	ethyl acetate extracts showed biocontrol activity against *R. solanacearum* (1 cm inhibition zone) and *F. oxysporum* (37.5% inhibition of mycelial growth). Both citrulline and chloramphenicol inhibited the growth of two microbial agents.	[226]
	aqueous	diuretic	albino rat	in vivo	oral administered	200, 400 mg/kg	showed significant urine output, increased Na^+^ and Cl^−^ excretion after 24 h, and significant decrease in K^+^ concentration in urine only at 6h	[227]
L, F, S	petroleum ether, alcohol and acetone	antimicrobial	bacteria (*K. pneumoniae*, *E. coli*, *S. typhi*, *B. cereus*)	in vitro	disk diffusion	5% *w/v* solution (extract), dissolving 250 mg (extract), with 5 mL of dimethyl formamide	showed high sensitivity to *K. pneumoniae* and *S. typhi*, moderate sensitivity to *E. coli*, and less sensitivity and resistance to *Bacillus cereus*.	[228]
F, S	methanol	larvicidal	*Ae. Aegypti*, *Anopheles stephensi*, *A. culicifacies and C. quinquefasciatus*		dose mortality	25–400 mg/L	significant mortality showed for the fruit at LC_50_ 51.6, 52.2, 118.3 and 157.1 mg/l while Seed at LC_50_ 66.9, 73.7, 123.8, 154.9 mg/l after 24 h	[229]
F, L (shoots), R	methanol, aqueous	antimicrobial	bacteria (*E. coli*, *S. aureus*, *S. typhi*, *P. aeruginosa*, *K. pneumonia*, *Enterococcus faecalis*, *Shigella flexnari*,and *B. cereus*)	in vitro		1–20 mg/mL	fruit showed more activity than shoot and root	[42]
L, S, R, F	aqueous	antimicrobial	bacteria (*S. typhi*, *E. coli*, *S. aureus*, *K. pneumonia*)	in vitro	agar well diffusion	500 mg/mL	susceptible against gram Ve^(-)^ bacteria were *S. typhi* leaf (2.5 cm), stem (2 cm), root (1.5 cm), fruit (1.4 cm), and *E. coli* leaf (2.2 cm), stem (3.3 cm), root (1.2 cm), fruit (1.6 cm) and inhibited the growth of Gram^(+)^ bacteria *S. aureus* stem (2.6 cm), *K. pneumonia* leaf (1 cm), stem (1 cm), root (1 cm), fruit (1.6 cm)	[230]
L, St, F, R	acetone, methanol	antioxidant		in vitro	FRAP		methanol extract of root (2153.3 mmol Fe(II)/mg extract) showed significantly (*p* < 0.05) higher ferric-reducing effect	[146]
				in vitro	DPPH		methanol extract of stem and root showed higher levels of free radical scavenging activity (IC50, 119.9 and 124.7 µg/mL	
				in vitro	ABTS		acetone extract of root observed the highest activity (20,195.9 µmol/g)	
L, St, R, F, Wp	ethanol, aqueous	immunomodulatory	albino rats	in vivo	delayed type hypersensitivity reaction, carbon clearance test and CCl_4_ induced oxidative stress model	200 mg/kg/day	significantly increased hypersensitivity, decreased carbon clearance and reduced oxidative stress, and exhibited maximum degree of immunomodulatory effect	[152]
St, L, F	petroleum ether, alcohol, and acetone	antimicrobial	bacteria (*E. coli*, *K. pneumoniae*, *S. typhi* and *B. cereus*)	in vitro	agar well diffusion method	250 mg (in 5 mL dimethyl formamide)	showed high sensitivity to *K. pneumoniae* and *S. typhi*, moderate sensitivity to *E. coli* and less sensitivity and resistance to *Bacillus cereus*	[231]
L, St, F, Fl	ethanol	antihemolytic	spectrophotometer method/human erythrocytes	in vitro	erythrocytes suspension	125, 250, 500, and 1000 μg/mL	hemolytic activity significantly increased in a dose-dependent manner	[232]
L, St, R	aqueous	antioxidant		in vitro	DPPH free radical scavenging	100, 200, 300, 400 and 500 μg	The highest percentage of antioxidants was shown at 500 μg con. 46.80 ± 0.58, 48.21 ± 0.82, and 44.30 ± 0.67 for leaf, stem and root	[233]
	aqueous, ethanol, methanol, petroleum ether	antimicrobial	bacteria (*E. coli*, *B. subtilis*, *S. aureus*, *K. pneumoniae*)	in vitro	agar well diffusion		aqueous leaf extract showed highest zone of inhibition against *B. subtilis* (29.62 mm); ethanolic leaf and stem extract showed min. inhibitory concentration (MIC) values of 15.0 μg/mL against B. subtilis and *S. aureus*	
L, F, Stb, R	petroleum ether, chloroform, acetone, ethanol and methanol	antioxidant		in vitro	DPPH assay	1, 2, and 5 mg/mL	methanolic stembark extract showed the highest antioxidant activity with the value of 0.323102 followed by 0.34188 (leaf, ethanol), 0.416667 (root chloroform), and 0.459242 (fruit ethanol)	[151]
	petroleum ether, chloroform, acetone, ethanol and methanol	antimicrobial	bacteria (*S. aureus*, *E. coli*, *K. pneumonia*, *P. aeruginosa*, *P. vulgaris*) fungi (*A. flavus*, *F. solani*, *R. stolnifer*), yeast (*S. cerevisiae*, *C. albicans*)	in vitro	disc diffusion	10 mg/mL	methanol root extract showed maximum antimicrobial activity against *P. aeruginosa* and *P. vulgaris* (17.67 ± 0.33 mm) while fruit extract (methanol) against P. aeruginosa (14.67 ± 0.33 mm), leaf extract (ethanol) against *P. vulgaris* (14 ± 0.58 mm) and stem bark (methanol) extract against *P. vulgaris* (14.67 ± 0.33 mm) were estimated	

Abbreviation: Wp—whole plant; Ap—aerial part; L—leaf; Fl—flower; F—fruit; S—seed; St—stem; Stb—stem bark; B—bark; R—root; BS—*β*-sitosterol. IC_50_—half-maximal inhibitory concentration; EC_50_—half maximal effective concentration; DPPH—2,2-diphenyl-1-picryl-hydrazyl-hydrate; pLDH—parasite lactate dehydrogenase; MTT—3-(4,5-dimethylthiazol-2-yl)-2,5-diphenyl-2H-tetrazolium bromide; TST—tail suspension test; FST—forced swim test; HepG2—Human liver cancer cell line; DMSO—Dimethyl sulfoxide; COX-1—Cyclooxygenase-1; COX-2—Cyclooxygenase-2; 15-LOX—Lipoxygenase; FRAP—Ferric Reducing Antioxidant Potential; ABTS—Azinobis-3-Ethylbenzthiazoline-6-Sulphonic acid; DPPH—2,2-Diphenyl-1-picrylhydrazyl; TDZ—Thidiazuron; NAA—Naphthalene acetic acid; BAP—Benzylaminopurine; sPLA2—Secretory phospholipase A2; AMA—adult motility assay.

**Table 5:** Biologically tested compounds of *S. surattense*.

Compound	Assay	Activity	Model/Cell Line	Result	Ref.
3-(4-hydroxy)-*N*-[2-(3-methoxyphenyl-4- hydroxyphenyl)-2- hydroxy]	in vitro/LPS-induced NO production assay	anti-inflammatory	RAW 264.7 cells	IC_50_ value of 12.23 ± 1.20 µM	[109]
*p*-hydroxy-phenylacetonitrile-*O*-(6′-*O*-acetyl)-*β*-*D*-glucopyranoside				IC_50_ value of 24.76 ± 1.97 µM	
caffeic acid	in vivo	neuroprotective	rat	significant activity through modulation of oxidative stress and neurochemical aspects	[112]
tribulusamide A	in vitro/MTT assay	hepatoprotective	D-gain/TNF-α-induced mouse hepatocytes	cytoprotective (97.2 ± 14.5% to 106.4 ± 10.1%) at low concentrations (10–20 µm) but cytotoxic at high concentration (50–200 µm) on D-gain/TNF-α-induced mouse hepatocytes	[106]
(7*R*,8*S*)-*threo*-glehlinoside C	in vitro/LPS-induced NO production assay	anti-inflammatory	RAW 264.7 cells	IC_50_ > 50 µM	[109]
2*Z*-(7*S*,8*R*)-aegineoside				IC_50_ 12.33 ± 1.21 µM	
(7*R*,8*R*)-3,5-dimethoxy-8′-carboxy-7′-en-3′,8-epoxy-7,4′-oxy-neolignan-4,9-diol				IC_50_ value of 19.69 ± 1.91, µM	
solamargine	in vitro/MTT assay	anti-tumoral	A549, hepg2	IC_50_ values of 15.7 ± 0.6 and 23.2 ± 0.8 µM	[105]
	in vitro/Sulforhodamine B cytotoxicity	anti-tumoral	human colon carcinoma cell line (HCT116)	strongly cytotoxic, but no induction of ccCK18 at cytotoxic doses > 10 mM	[134]
	in vitro/MTT assay	anti-tumoral	NIH-3T3 fibroblast cancer cells	showed strong cytotoxicity against 3T3 cell line with IC_50_ value of 7.55 ± 1.5	[163]
solasonine	in vivo/electrolyte flame photometry	antiurolithiatic	Albino rats	showed significant antiurolithiatic activity, with urine concentration ratio of 1.6	[213]
	in vitro/sulforhodamine B cytotoxicity	anti-tumoral	human colon carcinoma cell line (HCT116)	strongly cytotoxic, but no induction of ccCK18 at cytotoxic doses > 10 mM	[134]
diosgenin	in vitro/sulforhodamine B cytotoxicity	anti-tumoral	human colon carcinoma cell line (HCT116)	weakly cytotoxic (70–80% cell viability at 50 mM), and induced ccCK18 to 2-fold background levels	[134]
solasodine					
	in vivo/electrolyte flame photometry	antiurolithiatic	Albino rats	showed significant antiurolithiatic activity, with urine concentration ratio of 1.5	[213]
dioscine	in vitro/MTT assay	anti-tumoral	NIH-3T3 fibroblast cancer cells	showed strong cytotoxicity against 3T3 cell line with IC_50_ value of 3.3 ± 1.9 μg/mL	[163]
khasianine	in vitro/MTT assay	anti-tumoral	A549, MGC-803, hepg2	IC_50_ values of 26.7 ± 1.5, 35.4 ± 0.7, and 45.3 ± 2.1 µM	[105]
(22*R*, 25*R*)-16*β*-H-22*α*-N-spirosol-3*β*-ol-5-ene 3-*O-α*-L-rhamnopyran-osyl- (1 → 2)-[a-L-rhamnopyranosyl-(1 → 4)]-*β*-D-glucopyranoside	in vitro	anti-tumoral	A549, MGC-803, hepg2	IC_50_ values of 20.3 ± 1.1, 45.6 ± 1.5, and 26.1 ± 0.6 µM	[105]
(22*R*, 23*R*, 25*S*)-3*β*, 6*α*, 23-trihydroxy-5*α*-spirostane 6-*O-β*-*D*-xylopyranosyl-(1 → 3)-*O-β*-D-quinovopyranoside			A549, hepg2	IC_50_ values of 62.5 ± 1.6 and 88.8 ± 1.2 µM	
(22*R*, 23*S*, 25*R*)-3*β*, 6*α*, 23-trihydroxy-5α-spirostane 6-*O-β*-Dxylopyranosyl-(1 → 3)-*O-β*-D-quinovopyrano-side			A549	IC_50_ value of 71.2 ± 2.0 µM	
(22*R*, 23*S*, 25*S*)-3*β*, 6*α*, 23-trihydroxy-5*α*-spirostane 6-*O*-*β*-D-xylopyranosyl-(1 → 3)-*O*-*β*-D-quinovopyranoside			Mgc-803	IC_50_ value of 63.2 ± 0.8 µM	
solasaponin A	in vitro	anti-tumoral	A-549, hepg2	IC_50_ values of 8.51 ± 0.92 and 28.01 ± 2.72	[134]
solasaponin B				IC_50_ values of 10.52 ± 1.78 and 10.52 ± 1.48	
solasaponin C				IC_50_ values of 14.29 ± 3.21 and 16.38 ± 1.01	
solasaponin D	in vitro	anti-tumoral	A-549, hepg2	IC_50_ values of 9.44 ± 1.23 and 10.48 ± 1.23	
solasaponin E				IC_50_ values of 11.22 ± 1.21 and 4.82 ± 0.41	
solasaponin F				IC_50_ values of 12.35 ± 1.03 and >50	
solasaponin G				IC_50_ values of 37.82 ± 2.81 and 27.95 ± 3.02	
solasaponin H				IC_50_ values of 12.41 ± 2.66 and 22.03 ± 1.98	
xanthosaponin A	in vitro	anti-tumoral	MGC803, LN229, and SMMC7721	IC_50_ values of 40.24 ± 4.22, 69.43 ± 5.54, and 10.01 ± 1.12 µM	[137]
xanthosaponin B				IC_50_ values of 21.47 ± 3.02, 1186.25 ± 107.68, and 32.12 ± 3.14 µM	
carpesterol	in vitro	anti-diabetic	rat	potentially inhibited α-glucosidase activity with IC_50_ value of 42.26 ± 0.11 μM	[104]
oleanolic acid	in vivo	neuroprotective activity	rat	significant activity through modulation of oxidative stress and neurochemical aspects	[112]
cholesaponin A	in vitro/CCK-8 assay	anti-tumoral	A-549, hepg2, SMMC-7721, MGC-803, LN-229	IC_50_ (µM) values of 11.98 ± 2.02, 25.17 ± 3.24, 5.21 ± 0.47, 39.80 ± 3.77, and 8.83 ± 0.76	[135]
cholesaponin B				IC_50_ (µM) values of 6.33 ± 1.12, 4.50 ± 0.58, 5.71 ± 0.59, 2.81 ± 0.37, and 2.60 ± 0.36	
cholesaponin C				IC_50_ (µM) >100, 48.03 ± 3.37, >100, 36.82 ± 3.76, and 22.11 ± 2.53	
cholesaponin D				IC_50_ (µM) 7.41 ± 2.17, 18.23 ± 1.17, 4.67 ± 0.39, 24.75 ± 3.11, and 14.83 ± 1.65, respectively	
cholesaponin E	in vitro/CCK-8 assay	anti-tumoral	A-549, hepg2, SMMC-7721, MGC-803, LN-229	IC_50_ (µM) 10.09 ± 1.56, 28.23 ± 2.60, 12.52 ± 1.33, 13.49 ± 1.45, and 11.84 ± 0.98	[135]
cholesaponin F				IC_50_ (µM) 16.39 ± 2.82, 26.03 ± 2.93, 8.59 ± 0.90, 8.95 ± 0.97, and 6.16 ± 0.67	
(22*S*)-25[(*ß*-D-glucopyranosyl) oxy]-22-hydroxycholest-5-en-3*ß*-yl *O*-*α*-L rhamnopyrano-syl-(1 → 2)-*O*-[*α*-L-rhamnopyranosyl-(1 → 4)]-*ß*-D-glucopyran-oside				IC_50_ (µM) values of 56.03 ± 5.36, 27.23 ± 2.94, 3.22 ± 0.45, 21.76 ± 3.04, and 19.27 ± 2.14	
anguivioside XV				IC_50_ (µM) values of 12.64 ± 2.99, 25.36 ± 2.49, 4.59 ± 0.39, 13.58 ± 0.22, 7.89 ± 0.86	
gynuramide I	in vitro/LPS-induced NO production assay	anti-inflammatory	RAW 264.7 cells	inhibiting Nitric oxide production with IC_50_ value of 15.13 ± 1.36 µM	[142]
gynuramide II				inhibiting Nitric oxide production with IC_50_ value of 12.11 ± 1.20 µM	
gynuramide III				inhibiting Nitric oxide production with IC_50_ value of 15.61 ± 1.44 µM	
gynuramide IV				inhibiting Nitric oxide production with IC_50_ value of 14.17 ± 1.51 µM	
6″-*O*-acetyl soya-cerebroside I				inhibiting nitric oxide production with IC_50_ value of 41.99 ± 3.99 µM	
soya-cerebroside I				inhibiting Nitric oxide production with IC_50_ value of 43.86 ± 4.03 µM	
soya-cerebroside II				inhibiting Nitric oxide production with IC_50_ value of 48.66 ± 4.25 µM	
2*S*,3*S*,4*R*,8*E*-2-(2′*R*-2′-hydroxyhexacosanosylamino)-octadecene-1,3,4-triol				inhibiting nitric oxide production with IC_50_ value of 17.36 ± 1.83 µM	
methyl 9*S*,10*S*,11*R*-trihydroxy-12*Z*,15*Z*-octadecadienoate				inhibiting nitric oxide production with IC_50_ value of 44.17 ± 4.21 µM	
9*S*,10*S*,11*R*-trihydroxy-12*Z*,15*Z*-octadecadienoic acid				inhibiting nitric oxide production with IC_50_ value of 15.58 ± 1.58 µM	
methyl 9*S*,10*S*,11*R*-trihydroxy-12*Z*-octadecenoate				inhibiting nitric oxide production with IC_50_ value of 29.21 ± 2.91 µM	
9*S*,10*S*,11*R*-trihydroxy-12(*Z*)-octadecenoic acid				inhibiting nitric oxide production with IC_50_ value of 17.21 ± 0.89 µM	
methyl 9*S*,12*S*,13*S*-trihydroxyoctadeca-10*E*,15Z-dienoate				inhibiting Nitric oxide production with IC_50_ value of 42.72 ± 4.31 µM	
9*S*,12*S*,13*S*-trihydroxy-10*E*-octadecenoate				inhibiting Nitric oxide production with IC_50_ value of 27.56 ± 2.62 µM	
2′*S*-20-hydroxyl arachidic acid glycerol ester				inhibiting Nitric oxide production less with IC_50_ > 50 µM	
2′*S*-20-*O*-caffeoyl-20-hydroxyarachidic acid glycerol ester				inhibiting Nitric oxide production with IC_50_ value of 20.01 ± 1.99 µM	
2′*S*-22-*O*-caffeoyl-22-hydroxy-docosanoic acid glycerol ester				inhibiting Nitric oxide production with IC_50_ value of 15.31 ± 1.52 µM	
2′*S*-22-*O*-p-hydroxy-phenylpropionyloxy-22-hydroxy-docosanoic acid glycerol ester				inhibiting Nitric oxide production with IC_50_ value of 13.03 ± 1.32 µM	

## Supplementary Materials

The following supporting information can be downloaded at: https://www.mdpi.com/article/10.3390/ph17070948/s1, Table S1. Ethnomedicinal uses of *S. surattense*; Table S2. List of identified compounds found in *S. surattense*; Table S3. PRISMA Checklist with Detailed Information.
