# Supplementary material for: Ethnomedicinal Usage, Phytochemistry and Pharmacological Potential of Solanum surattense Burm. f
Source: Pharmaceuticals (Basel). 2024 Jul 15;17(7):948. doi: 10.3390/ph17070948 (PMC11280019; doi:10.3390/ph17070948)
Supplement: Supplementary file 1 [file pharmaceuticals-17-00948-s001.zip › pharmaceuticals-3043494-supplementary.pdf]

Supplimentary Table S1

Table S1. Ethnomedicinal uses of *S. surattense.*

Plant part	Country	Traditional medicinal uses	Mode of preparation/recipe	References
Wp	India	arthritis, asthma, blood complaints, bronchial asthma, cough, diarrhoea, dropsy, fever, haemorrhoids, leprosy, skin diseases, stomachache, throat diseases, urinary tract problems	decoction, juice, powder / oral administration	22 - 30
	Iran	antipyretic, asthma, bronchitis, cough, cold, inflammation skin and disease	-	31-32
	Nepal	leprosy, cough, dropsy	-	25
	Pakistan	abdomen pain, antipyretic, anthelmintic, asthma, blood purifier, breathing problems, chest congestions, colic pain, chronic constipation, cough, diuretic, fever, gas trouble, hair tonic, headache, jaundice, joint pain, kidney stones, leprosy, menstrual problem, paleness, rheumatism, skin problems, swelling, vaginal infection, wound healing,	ash, boiled, cooked as vegetable, decoction, oral administration, paste, topical application	19, 33 - 42
L	China	rheumatic pain	paste / topical application	43
	India	alopecia, cold, cough, respiratory diseases, dental problems, genitals prolapse, joint pain	mixture of leaf with honey / for massage, decoction (5g), juice with black pepper and honey, powder (50 g) with jaggery / oral administration, paste / topical application	44 - 52
	Pakistan	cough, joint pain, respiratory diseases, toothache, urinary disorders	paste and powder with honey / topical application, juice with the *Piper nigrum* seed powder, tincture, and decoction, /oral administration	53 - 57
	Sri Lanka	fever, cough, asthma	infusion and smoke / orally administration and inhalation	58
Fl	India	asthma, cough	fried / oral administration	30, 46

Plant part	Country	Traditional medicinal uses	Mode of preparation/recipe	References
	Pakistan	arthritis, cough	paste combined with egg white / for massage, powder with honey / oral administration	59 - 60
F	Bangladesh	tumor in humans, swelling of throat in cattle	maceration / oral administration	61
	China	sore throat	juice / oral administration	43
	India	aching ear, anthelmintic, anti-pyretic, anti-inflammatory, anti-asthmatic, cough, fever, fertility problems, joint pain, paralysis, stone bladder, toothache	juice / as ear drop, fresh or dried part, decoction, mixture with some oil / for massage, paste, smoke / inhalation, or oral administration	51-52**,** 62 - 69
	Nepal	toothache	dried part is chewed or directly placed, smoke / inhalation	69-70
	Pakistan	anti-gonorrhea, boils and pimples, broken organs, bronchial asthma, skin disorders, cough, diabetes, eye problems, fever, fertility problems, flu complication, gastrointestinal, headache, hemorrhoids, intestinal problems, liver disorders, migraine, musculoskeletal, obesity, pain, refrigerant, respiratory disease, scabies in animals, stomach disorders, toothache, would healing	powder, decoction/ with honey and powder, paste / topically applied, juice / applied on eye, oral administration / inhalation, fresh or dried part are mixed with something sweet, pulp / topical application	21, 53, 55 - 57, 60, 71 - 80
S	India	amenorrhea, antiseptic, dysmenorrhea, malaria, toothache	fruit pulp / oral administration, decoction / oral administration and gargle, vapor / inhalation	81 - 85
	Pakistan	cardiac disease, eye irritation, gastrointestinal problem, migraine, musculoskeletal, obesity, tooth infection,	Decoction, / rinsing and topical, seed with hot milk, fresh and dried part with honey, oral administration	33, 36, 55, 75, 78
St	Pakistan	indigestion, fever, cough, and asthma	decoction along with black pepper and salt / oral administration	86

Plant part	Country	Traditional medicinal uses	Mode of preparation/recipe	References
R	India	abdominal pain, arthritis, asthma, cough, fever, hemorrhoids, inflammatory, intestinal infection, weakness, jaundice, kidney problems, leprosy, measles, menstrual disorders, nervous system disorder, pain, smallpox, snake bite, toothache, urinary tract troubles	powder, formulation (Pf-9), decoction, formulation (Pf-6), powder along with *solanum indicum* linn., paste / topical application, powder (6g) with *Piper longum*, juice is taken, paste and tablet, and oral administration	30, 51 - 52, 63, 87 - 98
	Nepal	urinary troubles	powder with *Alternanthera sessilis* / oral administration	99
	Pakistan	appetite stimulant, asthma, cough, diabetes, fever, headache, intestinal worms, phlegmatic cough and fever, respiratory diseases, toothache	decoction (pf7), powder, decoction along with stem of *Tinospora cordifolia* / oral administration, used as a brush	21, 54, 56, 67, 71, 79, 100, 101
L and Fl	India	rheumatic pain	powder / topical	87
L and F	Pakistan	rheumatic pain	decoction / oral administration	88
Fl and F	Pakistan	chronic cough, discomfort, sore throat, body pains, fever, rheumatism, and chest complaints	juice mixed with honey / oral administration	89
St, Fl, and F	India	burning sensation	paste / topical	90
L, R, B, and F	Pakistan	toothache	decoction	91
L, St, and R	Pakistan	fever	decoction	92

[Pf = polyherbal formulation]

Supplimentary Table S2

Table S2. List of identified compounds found in *S. surattense*

Class of compounds	Compounds	Plant part	Ref.
Phenolics			
Phenolic amides			
1	*N*-*trans*-feruloyl tyramine	Wp	106
2	*N*-*p*-*trans*-coumaroyl tyramine	Wp	106
3	2-propenamide, *N*-[2-(dimethylamino)ethyl]-	L	107
4	Dihydro-*N*-feruloyltyramine	F	108
5	*N*-*trans*-coumaroyltyramine	F	108
6	*N*-*trans*-coumaroyloctopamine	F	108
7	*N*-[2-(3,4-dihydroxyphenyl)-2-hydroxyethyl]-3-(4-methoxyphenyl)-prop-2-enamide -	F	108
8	3-(4-hydroxy)-*N*-[2-(3-methoxyphenyl-4-hydroxyphenyl)-2- hydroxy]	F	109
Phenolic acids			
9	Ferulic acid	Wp	104
10	Evofolin B	Wp	106
11	Chlorogenic acid	L, F, St, R	110, 111
12	Caffeic acid	Ap	112
13	(1*R*,3*R*,4*R*,5*R*)-(-)-quinic acid	L	107, 113
14	2-Octylcyclopropene-1- Heptanol	L	107, 113
15	Eugenol	L, F	114
16	Methyl eugenol	F	114
17	(*E*)-isoeugenol	F	114
18	Butanedioic acid	F	115
Phenolic aldehydes			
19	Vanillin	L, S, F, R	114
Phenolic glycoside			

Class of compounds	Compounds	Plant part	Ref.
20	Chlorogenic acid ethyl ester-4′-*O*-*β*-*D*-glucopyranoside	F	109
21	Chlorogenic acid methyl ester-4′-*O*-*β*-*D*-glucopyranoside	F	109
22	*p*-hydroxyphenylacetonitrile-*O*-(6′-*O*-acetyl)-*β*-*D*- glucopyranoside	F	109
Flavonoids			
23	Apigenin	L, F, Pt, St, R	116, 111
24	Isoquercitrin	F	117
25	Gallocatechin	F	117
26	Catechin	F	117
27	Quercetin	F	117, 115
28	Flavone	F	117, 118
29	Luteolin	F	115
30	4H-1-benzopyran-4-one, 5,7-dihydroxy-2-(4-hydroxyphenyl)-3-methoxy-	F	115
31	5,7,4'-trihydroxy-8-methoxyflavone	F	108
32	5,7,4'-trihydroxy-6-methoxyflavone	F	108
33	5-hydroxy-8-methoxy-6,7-methylenedioxyflavone	F	108
34	7-hydroxy-6-methoxy coumarin	F	108
35	Fraxetin	F	108
36	5-hydroxy- 6,7,3',4'-tetramethoxyflavone	F	108
37	5-hydroxy-4',6,7-trimethoxyflavone	F	108
38	5,3'-dihydroxy-6,7,4'-tritermethoxyflavone	F	108
39	Acetovanillone	R	114
Coumarins			
40	Scopoline	L, F, R	119
41	Scopoletin	L, F, R	119
42	Esculin	L, F, R	119
43	Esculetin	L, F, R	119

**Class of compounds**	**Compounds**	**Plant part**	**Ref.**
Anthraquinones			
44	Emodin	L, St, R	111
Lignans			
45	*threo*-1-(4-hydroxy-3-methoxyphenyl)-2-{4-[(*E*)-3-hydroxy-1-propenyl]-2-methoxyphenoxy}-1,3-propanediol	Wp	106
46	Syringaresinol	Wp	106
47	Coniferol	WP	106
48	Simulanol	Wp	106
49	Balanophonin	Wp	106
50	Glycosmisic acid	Wp	106
51	Tribulusamide A	Wp	106
52	(7*R*,8*S*)-*threo*-glehlinoside C	F	109
53	2*Z*-(7*S*,8*R*)-aegineoside	F	109
54	(7*R*,8*R*)-3,5-dimethoxy-8′-carboxy-7′-en-3′,8-epoxy-7,4′- oxy-neolignan-4,9-diol	F	109
55	Glycerol α-guiacyl ether	F	115
Tannins			
56	Quinic acid	F	115
Alkaloids			
Quinoline alkaloids			
57	Isoquinoline	F	115
Steroidal alkaloids			
58	Solanoside A	Wp	120, 104
59	Solanoside B	Wp	120, 104
60	(22*R*,25*R*)-16α-H-22α-N-spirosol-3β-ol-5-ene-*O*-(3){α-L-rhamnopyranosyl-(1-2)-α-L rhamnopyranoside}-	Wp	104
61	Solanoid A	Wp	104

Class of compounds	Compounds	Plant part	Ref.
62	Solanoid B	Wp	104
63	Solamargine	Wp, Ap, F	104, 110, 121
64	Khasianine	Wp, Ap	104, 105, 122, 123, 124
65	Solasodine	F	125
66	(22*R*, 25*R*)-16*β*-H-22*α*-N-spirosol-3b-ol-5-ene 3-*O*-*α*-L-rhamnopyranosyl- (1 → 2)-[*α*-L-rhamnopyranosyl-(1→4)]-*β*-*D*-glucopyranoside	AP	105, 123, 124 122
67	Solasonine	Ap, L (shoot), F	105, 110, 121, 123, 124, 125, 126
68	Tomatidenol	Ap	105, 123, 124 122
69	Solanocarpine	S	127
70	Solanacarpigenin	S	127
71	Solanine	F	128
72	Solanidine	F	128
73	Solaxanine	F	129
74	Solasurine	F	110
75	*α*-solamargine	F	130
76	*ß*-solamargine	F	121
77	Solanearpidine	F	131
<u>*Terpenoids*</u>			
Monoterpenoids			
78	7*Z*-roseoside	F	132
79	Linalool	St	114
80	Camphor	St	114

Class of compounds	Compounds	Plant part	Ref.
81	*α*-terpineol	St	114
82	Geraniol	St, R	114
83	Isobornyl acetate	St, R	114
84	(*E*)-*β*-ionone	L, St	114
85	Dihydroactinidiolide	L	114
Sesquiterpenoids			
86	1-methylene-2b-hydroxymethyl-3,3-dimethyl-4b-(3methylbut-2enyl)- cyclohexene	L	113
87	1,3,3-trimethyl-2-hydroxymethyl-3,3-dimethyl-4–3-methylbut-2-enyl)- cyclohexene	L	113
88	dihydrophaseic acid 3′-*O*-*β*-*D*-glucopyranoside	F	132
89	Megastigmane	F	132
90	Matenoside A	F	132
91	Citroside A	F	132
92	Byzantionoside B	F	132
93	Actinidioionoside A	F	132
94	Melongenaterpene C15-*O*-*β*-*D*-glucopyranoside	F	132
95	(9Z)-3,7,11,15-tetramethyl -hexadeca-1,6,10-triene-3,5,14,15-tetraol-5-*O*-*β*-*D*-glucopyranoside	F	132
96	*α*-Cubebene	F,R	114
97	*β*-Cubebene	F, R	114
98	(*E*)-*β*-farnesene	F	114
99	*trans*-muurola-4(14),5-diene	F, R	114
100	*epi*-cubebol	L, F, R	114
101	Indipone	St	114
102	*α*-bulnesene	L	114
103	Cubebol	L, F, R	114
104	*trans*-calamenene	St, R	114
105	*δ*-cadinene	F	114

Class of compounds	Compounds	Plant part	Ref.
106	β-sesquiphellandrene	L	114
107	α-cadinene	F	114
108	α-copaen-11-ol	F	114
109	β-sesquiphellandrene	L	114
110	α-cadinene	F	114
111	α-copaen-11-ol	F	114
112	α-calacorene	L,St, F, R	114
113	β-calacorene	St, R	114
114	Cypertundone	L	114
115	α-bisabolol	L, St	114
116	Germacra-4(15),5,10(14)-trien-1α-ol	R	114
117	Amorpha-4,9-dien-2-ol	R	114
118	Cadalene	F, St	114
119	14-hydroxy-9-*epi*-(*E*)-caryophyllene	L	114
120	*trans*-calamenen-10-ol	R	114
121	*cis*-calamenen-10-ol	F, R	114
122	α-cadinol	F, R	114
123	Pogostol	L	114
124	15-copaenol	R	114
125	Cubenol	R	114
126	τ-muurolol (torreyol)	L, F	114
127	Caryophylla-4(12),8(13)-dien-5-ol	L	114
128	*cis*-cadin-4-en-7-ol	St, L	114
129	Humalane-1,6-dien-3-ol	R	114
130	Muurola-4,10(14)-dien-1β-ol	L	114
131	1-*epi*-cubenol	F, R	114
132	1,10-di-*epi*-Cubenol	St	114

Class of compounds	Compounds	Plant part	Ref.
133	Ledol	R	114
134	Caryophyllene oxide	L, R	114
135	Spathulenol	L	114
136	Caryophyllenyl alcohol	L	114
137	(*E*)-nerolidol	L, F	114
138	(2*Z*,6*E*)-farnesol	F	114
139	Cyclocolorenone	R	114
140	14-oxy-*α*-muurolene	R	114
141	Solavetivone	R	114
142	Octadecane	L, F	114
143	5-hydroxy-*cis*-calamenene	F	114
144	Nootkatone	R	114
145	6,10,14-trimethyl-2-pentadecanone	L, F	114
146	3-hydroxysolavetivone	R	114
Diterpenoids			
147	Phytol (3,7,11,15-tetramethyl-2-hexadecane-1-ol)	L	107, 113, 114
148	Neophytadiene	L	114
149	(*E,E*)-geranyllinalool	F	114
150	Lycopene	F	115
151	Carotenoids	F	115
Triterpenoids			
152	26-*O*-*β*-D-glucopyranosyl-(25*R*)-furosta-5-ene-3-*β*-yl-*O*-*α*-L-rhamnopyranosyl-(1″-2′)-*O*-*α*-L-rhamnopyranosyl-(1‴-3″)-*O*-*β*-D-glucopyranoside	F, S	133
153	Dioscin	Wp	104
154	22*R*, 23*S*, 25*R*)-3*β*, 6*α*, 23-trihydroxy-5*α*-spirostane 6-*O*-*β*-dxylopyranosyl-(1→3)-*O*-*β*-*D*-quinovopyranoside	Ap	105

Class of compounds	Compounds	Plant part	Ref.
155	(22*R*, 23*S*, 25*S*)-3*β*, 6*α*, 23-trihydroxy-5*α*-spirostane 6-*O*-*β*-*D*-xylopyranosyl-(1→3)-*O*-*β*-*D*-quinovopyranoside	Ap	105
156	(22*R*, 23*R*, 25*S*)-3*β*, 6*α*, 23-trihydroxy-5*α*-spirostane 6-*O*-*β*-*D*-xylopyranosyl-(1→3)-*O*-*β*-*D*-quinovopyranoside	Ap	105
157	Torvoside K	Ap	105
158	Torvoside J	Ap	105
159	Torvoside L	Ap	105
160	Aculeatiside A	Ap	105
161	Solasaponin A	F	134
162	Solasaponin B	F	134
163	Solasaponin C	F	134
164	Solasaponin D	F	134
165	Solasaponin E	F	134
166	Solasaponin F	F	134
167	Solasaponin G	F	134
168	Solasaponin H	F	134
169	16,26-epoxy-furostanol	F	134
170	Furostanol	F	135
171	Isospirostanol	F	135
172	Pseudo-spirostanol	F	135
173	Diosgenin	F, St	136, 126
174	Xanthosaponin A	F	137
175	Xanthosaponin B	F	137
176	26-*O*-(*β*-*D*-glucopyranosyl) nuatigenin 3-*O*- *α*-L-rhamnopyranosyl-(1→4)-*β*-*D*-glucopyranoside	Ap	105
177	Carpesterol	Wp, F, S	121, 131, 104, 127

Class of compounds	Compounds	Plant part	Ref.
178	Stigmasterol	Ap, F	131, 121, 122, 114
179	Campesterol	L, F, St, R	121, 111 138, 114
180	Ergosterol	L, F, St, R	111
181	Withanolide B	L, F, St, R	127 111
182	*β*-sitosterol	Ap, L, St, R	122, 111,114
183	3-oxo-oleane-9(11)-ene	Ap	122,112
184	Lupeol acetate	Ap	122, 112
185	Lupa-12(13),20(29)-diene-3-one	Ap	122, 112;
186	Oleanolic acid	Ap, L, F, St, R	111, 112, 107, 122, 114
187	Lupeol	L, F, St, R	111, 107, 114
188	Ursolic acid	L, F, St, R	111, 107
189	*Trans*-squalene	L	111, 107
190	Cycloartenol	F	121
191	Cholesterol	F	121
192	Sitosteryl glucoside	F	121
193	Stigmasteryl glucoside	F	121
194	Cycloartenol	F	121
195	Estriol	F	114
196	Estradiol	F	114
197	3*β*-hydroxy-5 alpha-cholest-8-en-7-one	F	114
198	4*α*-methyl-24*ξ*-ethyl-5*α*-cholest-7-en-3*β*,22*ξ*-diol	F	139
199	3*β*,22*ξ*-dihydroxy-4*α*-methyl-24*ξ*-ethyl-5*α*-cholest-7-en-6-one	F	139
200	3*β*-benzoxy-l4*β*,22*ξ*-dihydroxy-4*α*-methyl-24*ξ*-ethyl 5*α*-cholest-7-en-6-one	F	139
201	3*β*-benzoxy-14*α*,22*ξ*-dihydroxy-4*α*-methyl-24*ξ* ethyl-5*α*-cholest-7-en-6-one	F	139
202	3*β*-(*p*-hydroxy)- benzoxy-22*ξ*-hydroxy-4*α*-methyl-24*ξ*-ethyl-5*α*-cholest-7-en-6-one	F	139

**Class of compounds**	**Compounds**	**Plant part**	**Ref.**
203	Cholesaponin A	F	135
204	Cholesaponin B	F	135
205	Cholesaponin C	F	135
206	Cholesaponin D	F	135
207	Cholesaponin E	F	135
208	Cholesaponin F	F	135
209	(22*S*)-25[(*β*-*D*-glucopyranosyl)oxy]-22-hydroxycholest-5-en-3*β*-yl-*O*-*α*-L rhamnopyranosyl-(1→2)-*O*-[*α*-L- rhamnopyranosyl-(1→4)]-*β*-*D*-glucopyranoside	F	135
210	Anguivioside XV	F	135
211	Huzhangoside C	F	132
212	Taraxerol	F	132
213	(*E*)-4,8-dimethyl,1,3,7-nonatriene	F	115
214	Squalene	F, R	115
Lipids			
Fatty acids			
215	Palmitic acid	Wp, St, F, R	140, 141, 114, 115
216	Linoleic acid	Wp, St, R	140, 141, 114
217	Oleic acid	Wp, St, F	140 141, 104, 114
218	Ethyl hexanoate	L	107
219	Ethyl hexadecanoate	L	107
220	Hexadecanoic acid	L	107, 113
221	Dodecanoic acid	L, St, R	107, 114
222	Tetradecanoic acid	L, St, R	107, 114
223	9,12,15-octadecatrienoic acid, (*Z*,*Z*,*Z*)-	L	107
224	Octadecanoic acid	L	107
225	Hexadecanoic acid, 2-hydroxy-1- (hydroxymethyl) ethyl ester	L	107

**Class of compounds**	**Compounds**	**Plant part**	**Ref.**
226	Diisooctyl phthalate	L	107
227	Arachidic acid	S	141
228	Stearic acid	S	141
229	Nonanoic acid	F, St,R	114
230	Decanoic acid	St, R	114
231	*cis*-4-Hydroxydodec-6-enoic acid lactone	L, St	114
232	Pentadecanoic acid	St	114
Fatty aldehydes			
233	Nonanal	F	114
234	(2*E*,4*E*)-decadienal	R	114
235	Dodecanal	St	114
236	Tridecanal	F	114
237	Tetradecanal	F, L	114
238	Pentadecanal	F, L, St	114
239	Hexadecanal	F	114
240	9,12,15-octadecatrienal	L	114
241	Tetracosanal	F	114
242	Pentacosanal	F	114
243	Hexacosanal	F	114
Fatty alcohols			
244	1-octanol	St	114
245	(6*Z*)-nonenol	St	114
246	(*Z*)-dihydroapofarnesol	F	114
Fatty amide			
247	(9*Z*)-octadecenamide	St	114
248	Octadecanamide	St	114

Class of compounds	Compounds	Plant part	Ref.
Sphingolipids			
249	6″-*O*-acetyl soya-cerebroside I	F	142
250	Soya-cerebroside I	F	142
251	Soya-cerebroside II	F	142
252	2*S*,3*S*,4*R*,8*E*-2-(2′*R*-2′-hydroxyhexacosanosylamino)-octadecene-1,3,4-triol	F	142
253	Gynuramide I	F	142
254	Gynuramide II	F	142
255	Gynuramide III	F	142
256	Gynuramide IV	F	142
Oxylipins			
257	Methyl 9*S*,10*S*,11*R*-trihydroxy-12*Z*,15*Z*-octadecadienoate	F	142
258	9*S*,10*S*,11*R*-trihydroxy-12*Z*,15*Z*-octadecadienoic acid	F	142
259	Methyl 9*S*,10*S*,11*R*-trihydroxy-12*Z*-octadecenoate	F	142
260	9*S*,10*S*,11*R*-trihydroxy-12(*Z*)-octadecenoic acid	F	142
261	Methyl 9*S*,12*S*,13*S*-trihydroxyoctadeca-10*E*,15*Z*-dienoate	F	142
262	9*S*,12*S*,13*S*-trihydroxy-10*E*-octadecenoate,	F	142
263	2′*S*-20-hydroxyl arachidic acid glycerol ester	F	142
Phenolic lipids			
264	2′*S*-20-*O*-caffeoyl-20-hydroxy arachidic acid glycerol ester	F	142
265	2′*S*-22-*O*-caffeoyl-22-hydroxy-docosanoic acid glycerol ester	F	142
266	Phenyl propionyloxy-22-hydroxy-docosanoic acid glycerol ester	F	142
Alcohols			
267	2-phenylethyl alcohol	St	114
268	n-pentadecanol	F	114
269	n-hexadecanol	L	114

Class of compounds	Compounds	Plant part	Ref.
270	Octadecanol	L	114
271	1,2-bis(4-hydroxy-3-methoxyphenyl)-1,3-propanediol	Wp	106
272	1,2-ethanediol	F	114
Polyalcohol			
273	Xylitol	F	114
Sugars			
274	Glucose	F	131
275	Rhamnose	F	131
276	Galactose	F	131
277	Hexose	F	131
278	Sucrose	F	114
279	Methyl *α-D*-galacto-pyranoside	F	142
Esters			
280	Methyl caffeate	Wp, F	104, 110
281	Ethyl oleate	F	114
282	Methyl (*Z*,*Z*)-9,12-octadecadienoate	F	114
283	Methyl hexadecanoate	F	114; 118
284	Methyl propanoate	F	114
285	Methyl tetradecanoate	F	118
286	9-methyl-10,12-hexadecadien-1-ol acetate	F	118
287	Methyl (*Z*)-pentadec-8-enoate	F	118
288	Methyl (2*E*)-2,3-dimethyl-2-hexadecenoate	F	118
289	Methyl eicosa- 5,8,11,14,17-pentaenoate	F	118
290	1-octadecene	F	118
291	9-eicosene	F	118
292	1-tricosene	F	118

Class of compounds	Compounds	Plant part	Ref.
293	3-eicosene	F	118
294	*E*-2-octenyl tiglate	F	118
295	(*Z*)-9-methyl octadecenoate	F	118
296	γ-nonalactone	St	114
297	γ-dodecalactone	F	114
298	Acetyl tributyl citrate	R	114
299	*cis*-Methyl jasmonate	L	114
300	Methyl palmitate	L, St, F, R	114
301	Methyl linoleate	L, St, R	114
302	Methyl linolenate	S, R	114
303	Methyl ricinoleate	L, St, R	114
304	Ethyl palmitate	F	114
305	Methyl stearate	R	114
306	Ethyl oleate	F	114
307	*cis*-3-Hexenyl benzoate	L	114
308	Benzyl benzoate	L, F	114
309	Isopropyl myristate ester	St	114
Hydrocarbon			
310	2-tridecanone	L	114
311	Tetracosane	L, St, F, R	114
312	Hexadecane	F	114
313	Heptadecane	F	114
314	1-octadecene	F	114
315	Nonadecyne	L	114
316	1-nonadecene	F	114
317	Nonadecane	St, F	114
318	Eicosane	L, St, F	114

Class of compounds	Compounds	Plant part	Ref.
319	Heneicosane	L, F	114
320	Docosane	L, F, R	114
321	(*Z*)-9-tricosene	F	114
322	1-tricosene	F	114
323	Tricosane	L, St, F, R	114
324	1-tetracosene	F	114
325	Tetracosane	L, F	114
326	1-pentacosene	L, F	114
327	Pentacosane	L, St, F, R	114
328	Hexacosane	L, St	114
329	Heptacosane	L, St, F, R	114
330	3-methylheptacosane	L, St	114
331	Octacosane	L, St	114
332	2-methyloctacosane	L, St	114
333	Nonacosane	L, St	114
334	3-methylnonacosane	L, St	114
335	2-methyltriacontane	L, St	114
336	Untriacontane	L, St	114
Vitamins			
337	Vitamin C (ascorbic acid)	L, F, S	43, 114
338	Vitamin E	L	107

Ap - aerial part, F - fruit, Fl - flower, Pt - petals, L - leaf, S - seed, St - stem, Stb - stem bark, Wp - whole plant

Supplimentary Table S3

Table S3: PRISMA Checklist with Detailed Information

Section/Topic	Item No	Checklist Item	Description	Reported on Page
**TITLE**				
Title	1	Identify the report as a systematic review, meta-analysis, or both.	The title identifies the document as a systematic review.	1
**ABSTRACT**				
Structured summary	2	Provide a structured summary including, as applicable: background, objectives, data sources, study eligibility criteria, participants, interventions, study appraisal and synthesis methods, results, limitations, conclusions and implications of key findings, systematic review registration number.	The abstract provides a structured summary covering the background, objectives, methods, results, and conclusions.	2
**INTRODUCTION**				
Rationale	3	Describe the rationale for the review in the context of what is already known.	The rationale for reviewing Solanum surattense is provided, emphasizing its traditional medicinal uses and pharmacological potential.	3-5
Objectives	4	Provide an explicit statement of the questions being addressed with reference to participants, interventions, comparisons, outcomes, and study design (PICOS).	The objectives of the review are clearly stated, focusing on the ethnomedicinal uses, chemical constituents, and pharmacological activities of Solanum surattense.	5
**METHODS**				
Protocol and registration	5	Indicate if a review protocol exists, if and where it can be accessed (e.g., Web address), and, if available, provide registration information including registration number.	No specific protocol registration mentioned.	Not Reported

Eligibility criteria	6	Specify study characteristics (e.g., PICOS, length of follow-up) and report characteristics (e.g., years considered, language, publication status) used as criteria for eligibility, giving rationale.	The study characteristics and inclusion criteria are specified, including types of studies, languages, and publication years.	6-7
Information sources	7	Describe all information sources (e.g., databases with dates of coverage, contact with study authors to identify additional studies) in the search and date last searched.	Information sources include B-on, Google Scholar, PubMed, Science Direct, and Web of Science. The dates of coverage and the last search date are provided.	6-7
Search	8	Present full electronic search strategy for at least one database, including any limits used, such that it could be repeated.	The electronic search strategy is described, including keywords and database-specific search terms.	6-7
Study selection	9	State the process for selecting studies (i.e., screening, eligibility, included in systematic review, and, if applicable, included in the meta-analysis).	The process of study selection, including screening and eligibility criteria, is detailed.	6-7
Data collection process	10	Describe method of data extraction from reports (e.g., piloted forms, independently, in duplicate) and any processes for obtaining and confirming data from investigators.	The data extraction process is described, including the use of piloted forms and independent extraction by multiple reviewers.	6-7
Data items	11	List and define all variables for which data were sought (e.g., PICOS, funding sources) and any assumptions and simplifications made.	The data items collected, including variables such as chemical constituents and pharmacological activities, are listed.	7
Risk of bias in individual studies	12	Describe methods used for assessing risk of bias of individual studies (including specification of whether this was done at the study or outcome level), and how this information is to be used in any data synthesis.	Methods for assessing the risk of bias in individual studies are described, including assessment tools and criteria used.	7
Summary measures	13	State the principal summary measures (e.g., risk ratio, difference in means).	The principal summary measures used in the review are stated.	7
Synthesis of results	14	Describe the methods of handling data and combining results of studies, if done, including	Methods for data synthesis and combining study results, including consistency measures, are described.	7

		measures of consistency (e.g., I^2) for each meta-analysis.		
Risk of bias across studies	15	Specify any assessment of risk of bias that may affect the cumulative evidence (e.g., publication bias, selective reporting within studies).	The assessment of risk of bias across studies is specified, including potential publication bias and selective reporting.	7
Additional analyses	16	Describe methods of additional analyses (e.g., sensitivity or subgroup analyses, meta-regression), if done, indicating which were pre-specified.	Methods for additional analyses, including sensitivity and subgroup analyses, are described.	7
**RESULTS**				
Study selection	17	Give numbers of studies screened, assessed for eligibility, and included in the review, with reasons for exclusions at each stage, ideally with a flow diagram.	The numbers of studies screened, assessed for eligibility, and included in the review are provided, with reasons for exclusions. A flow diagram is included.	8
Study characteristics	18	For each study, present characteristics for which data were extracted (e.g., study size, PICOS, follow-up period) and provide the citations.	The characteristics of included studies, including study size, participants, interventions, and outcomes, are presented.	8-14
Risk of bias within studies	19	Present data on risk of bias of each study and, if available, any outcome level assessment (see item 12).	Data on the risk of bias within each study are presented.	14
Results of individual studies	20	For all outcomes considered (benefits or harms), present, for each study: (a) simple summary data for each intervention group (b) effect estimates and confidence intervals, ideally with a forest plot.	Results for individual studies, including summary data and effect estimates, are presented.	14-20
Synthesis of results	21	Present results of each meta-analysis done, including confidence intervals and measures of consistency.	Results of meta-analyses, including confidence intervals and measures of consistency, are presented.	20-22
Risk of bias across studies	22	Present results of any assessment of risk of bias across studies (see Item 15).	Results of assessments of risk of bias across studies are presented.	22
Additional analysis	23	Give results of additional analyses, if done (e.g., sensitivity or subgroup analyses, meta-regression [see Item 16]).	Results of additional analyses are presented.	22-23

**DISCUSSION**				
Summary of evidence	24	Summarize the main findings including the strength of evidence for each main outcome; consider their relevance to key groups (e.g., healthcare providers, users, and policymakers).	The main findings are summarized, including the strength of evidence for each outcome.	23-25
Limitations	25	Discuss limitations at the study and outcome level (e.g., risk of bias), and at the review level (e.g., incomplete retrieval of identified research, reporting bias).	Limitations at both the study and review levels are discussed.	25
Conclusions	26	Provide a general interpretation of the results in the context of other evidence, and implications for future research.	General interpretation of the results in the context of other evidence and implications for future research are provided.	25-26
**FUNDING**				
Funding	27	Describe sources of funding for the systematic review and other support (e.g., supply of data); role of funders for the systematic review.	The sources of funding and other support for the systematic review are described.	Not Reported

This detailed PRISMA checklist provides comprehensive coverage of all required elements for a systematic review, ensuring clarity and transparency in reporting.
